# Efficient red circularly-polarized phosphorescence from pyrene derivatives mediated by locked axial chirality scaffold

**DOI:** 10.1039/d6sc01341d

**Published:** 2026-05-05

**Authors:** Wenbin Huang, Chenlong Wei, Meng Wang, Qian Zhang, Zikai He

**Affiliations:** a College of Frontier Sciences, Harbin Institute of Technology Shenzhen 518055 China hezikai@hit.edu.cn

## Abstract

As a classic polycyclic aromatic hydrocarbon luminophore, pyrene exhibits considerable potential for optoelectronic applications yet faces challenges in achieving chiral luminescence, particularly long-lived red circularly polarized phosphorescence. In this work, we report a strategy for manipulating the axial chirality of binaphthalene to enable pyrene to achieve efficient circularly polarized luminescence and red circularly polarized phosphorescence. It is revealed that the locked axial chiral binaphthalene not only boosts overall intersystem crossing *via* its transient triplet excited state but also amplifies circularly polarized phosphorescence through mediating structural rigidity and transition dipole moments. Bright circularly polarized luminescence with a high quantum yield of 65.7% and distinct red circularly polarized phosphorescence with a dissymmetry factor of 6.5 × 10^−3^ and a persistent lifetime of 381.9 ms, are obtained. The outlined structure-property relationship provides insights into the design principle for developing efficient circularly polarized luminescent materials.

## Abstract 2

The work presents a strategy to achieve circularly polarized fluorescence and phosphorescence with enhanced intersystem crossing and amplified the dissymmetry factor of pyrene derivatives by employing locked axially chiral binaphthalene.

## Graphical abstract

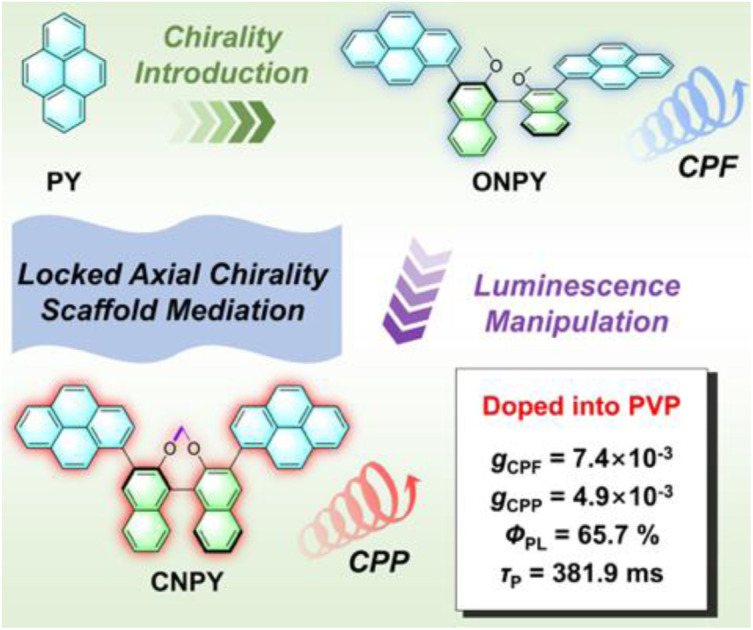

## Introduction

Red circularly polarized phosphorescence (CPP) is a highly desirable property for biological imaging, environmental sensing, and three-dimensional displays^1–4^ benefiting from its synergistic advantages of deep tissue penetration, unique chiral optics, and long-lived afterglow.^5–11^ However, achieving efficient red CPP remains a formidable challenge.^1,12–14^ First, the inherently small energy gap between the triplet excited state (T_1_) and the ground state (S_0_) for red-light emission promotes nonradiative decay, leading to severe phosphorescence quenching.^15^ Second, the large energy gap between the singlet excited state (S_1_) and the T_1_ state hinders efficient intersystem crossing (ISC), which is essential for populating the phosphorescent triplet state.^16–18^ Third, in chiral systems, flexible molecular frameworks often lead to rapid relaxation of chiral excited states, thereby weakening circularly polarized light emission.^19–22^ These fundamental limitations collectively impede the development of high-performance red CPP materials, necessitating the exploration of novel molecular design strategies.^23–29^

Pyrene, a typical polycyclic aromatic hydrocarbon luminophore, has attracted significant attention as a building block for developing luminescent materials due to its high fluorescence quantum yield and tunable π-conjugated planar structure.^30^ However, the lack of chirality in pyrene limits its applications in chiral optoelectronics, for instance, circularly polarized emission.^31^ Additionally, pyrene-based materials usually exhibit strong fluorescence but weak phosphorescence, which limits their applications in bioimaging and information encryption that require long-lived luminescent signals.^32–36^ Thus, several strategies have been attempted to introduce chirality into pyrene derivatives, including attaching chiral side chains^37–41^ and constructing chiral supramolecular assemblies,^42–45^ but these approaches often suffer from weak chiral influence, poor stability of chiral structure, inefficient ISC, and inferior phosphorescence.^46–48^ For example, chiral side chain modification introduces weak chiral induction, resulting in a low dissymmetry factor. Chiral supramolecular assemblies rely on non-covalent interactions, which are sensitive to external conditions and exhibit poor structural stability and reproducibility. Meanwhile, these strategies can only introduce chirality but cannot simultaneously improve ISC efficiency and phosphorescence performance. This urgently necessitates the development of novel molecular construction strategies to overcome the limitations.

**Scheme 1:**
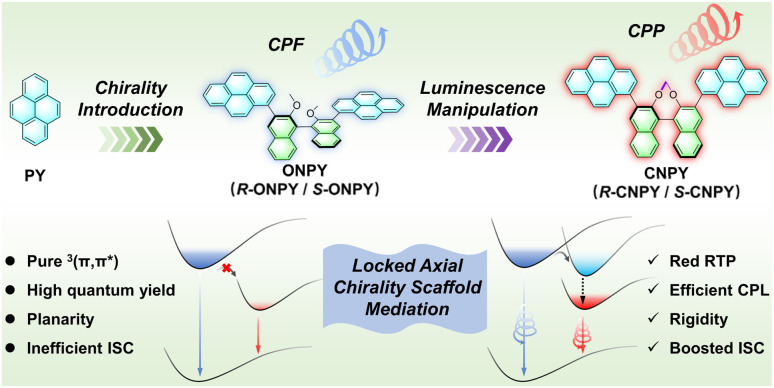
Diagram of molecular structural engineering and strategy for manipulating the locked axial chirality of binaphthalene to enable pyrene efficient circularly-polarized fluorescence (CPF) and red circularly-polarized phosphorescence.

Herein, we have successfully developed a novel strategy to achieve efficient circularly polarized luminescence (CPL) and red CPP from two pyrene derivatives (ONPY and CNPY) by manipulating the axial chirality of the binaphthalene skeleton (Scheme 1). By investigating the photophysical properties of pyrene, binaphthalene, ONPY, and CNPY, we systematically depicted the effects of open and locked axial chirality on the luminescent behavior of pyrene derivatives. They not only enhance intersystem crossing *via* the mediation of the transient triplet excited state of the chiral center but also amplify chiral emission by fixing the relative orientation of the pyrene and binaphthalene moieties and reducing conformational changes. Specifically, when doped into poly(vinyl pyrrolidone) (PVP), CNPY exhibits bright CPL with a |*g*_lum_| of 7.4 × 10^−3^ and a *Φ*_PL_ of 65.7%, as well as distinct red-CPP with a |*g*_lum_| of 6.5 × 10^−3^ and a *τ*_P_ of 381.9 ms.

## Results and discussion

### Molecular design and synthesis

Two derivatives were designed and synthesized: open axially chiral naphthalene-pyrene scaffold (ONPY) and locked axially chiral naphthalene-pyrene scaffold (CNPY).^49,50^ The difference lies in the presence of a bridging group in CNPY that locks the axial chirality of the binaphthalene unit, while ONPY has no such bridging group, resulting in a flexible axial chirality (Scheme 1). The pure enantiomers, namely, *R*-ONPY, *S*-ONPY, *R*-CNPY, and *S*-CNPY, were prepared from commercially pure binaphthalene starting materials (Scheme S1, SI). The synthetic procedures and the corresponding structural characterization (^1^H NMR, ^13^C NMR, high-resolution mass spectrum) details are summarized in the SI. The chiral high-performance liquid chromatography show that all enantiomers achieve separation, and the optical purity of each enantiomer is greater than 99% (ee > 99%) (Chart S7, SI).

### Photophysical properties

**Fig. 1:**
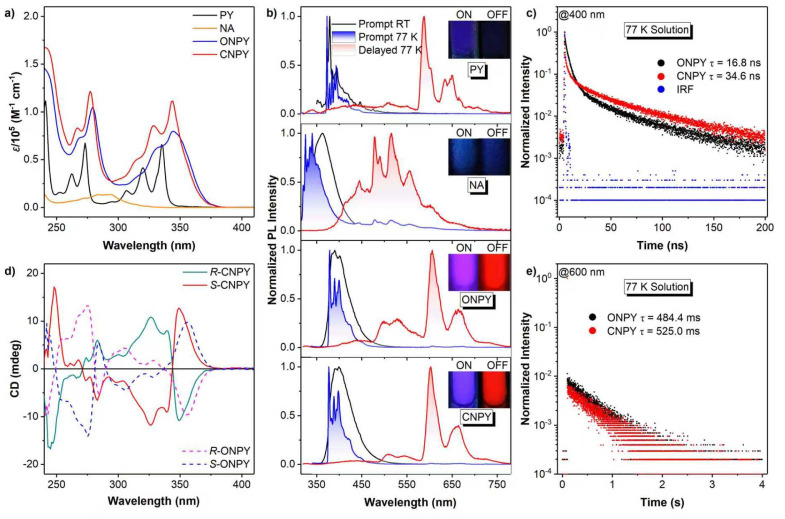
(a) UV-visible absorption spectra of pyrene (PY), binaphthalene (NA), ONPY, and CNPY solutions (10^−5^ M) at room temperature (RT); (b) normalized prompt PL spectra in solution at RT, and normalized prompt PL and delayed (1 ms) spectra in solution at 77 K for PY, NA, ONPY and CNPY, respectively; time-resolved (c) fluorescence and (e) phosphorescence decay curves and their respective lifetimes for ONPY and CNPY in solution at 77 K; (d) circular dichroism spectra in solution at RT for *R*-CNPY, *S*-CNPY, *R*-ONPY, *S*-ONPY, respectively.

The UV-visible absorption spectra in Fig. 1a reveal distinct electronic transitions. Pyrene (PY) exhibits characteristic absorption bands at 300–350 nm, corresponding to the π–π* transition of S_0_ → S_2_ based on its conjugated aromatic backbone.^51^ Since the S_0_ and S_1_ states have the same symmetry, transitions between states with identical symmetry are forbidden in accordance with the symmetry selection rule for electronic transitions, and thus the S_0_ → S_1_ absorption band cannot be detected. For ONPY and CNPY, the absorption bands corresponding to PY segments bathochromically shift to 300–380 nm due to increased molecular π-conjugation with binaphthalene (NA), with CNPY showing a more pronounced enhancement of absorption coefficient but a slightly higher-energy absorption edge than that of ONPY. It indicates that the locked skeleton in CNPY induces molecular rigidification and conjugation from the binaphthalene framework to the pyrene moieties, leading to an improved Frank–Condon factor.

The photoluminescence (PL) spectra in Fig. 1b provide insights into photophysical properties. At room temperature in 2-MeTHF solutions, PY and NA emit deep blue fluorescence with peaks at 378 nm and 363 nm, respectively, whereas ONPY and CNPY show red-shifted fluorescence emission peaks at 390 nm and 392 nm, respectively. Upon cooling to 77 K (frozen matrix), all luminophores retain prompt fluorescence with more distinct vibrational profiles. The nanosecond lifetimes monitored at 400 nm proved their fluorescence nature. Several new red-shifted emission bands emerge, spanning 570 to 750 nm for PY, 380 to 700 nm for NA, and 380 to 750 nm for ONPY and CNPY, respectively. They are attributed to phosphorescence based on their millisecond-decay curves in Fig. 1e. Notably, the phosphorescence of ONPY and CNPY is primarily derived from the pyrene fragment (575–700 nm) and secondarily from the binaphthalene fragment (475–575 nm), as identified by the wavelength and vibrational characteristics of their respective spectral profiles, while their fluorescence is entirely derived from the pyrene fragment. Moreover, compared to PY, ONPY and CNPY exhibit much more intense and persistent red phosphorescence, suggesting that superior intersystem crossing and phosphorescence efficiency are achieved in ONPY and CNPY. PL performance was successfully mediated by the chiral center, as evidenced by the observed phosphorescence from the binaphthalene segment. The longer fluorescence and phosphorescence lifetimes of CNPY indicate that the locked structure enhances structural rigidity, thereby weakening nonradiative transitions and further improving the luminescent performance.

The chiroptical properties of pure enantiomers are first characterized by circular dichroism (CD) spectroscopy. As shown in Fig. 1d, all chiral enantiomeric derivatives exhibit mirror-imaged CD signals. Distinct alternating positive and negative cotton effects are observed at 240–380 nm, which match well with their absorption bands. Careful investigation of the intensity and direction of CD signals reveals that the axially chiral binaphthalene skeleton induces obvious chiroptical absorption behaviours in pyrene segments. Meanwhile, the locked structure results in a higher CD intensity. The calculated *g*_CD_ of the CNPY enantiomers is higher than that of ONPY (Fig. S2, SI). It indicates that in the ground state, the flexible configuration in ONPY results in a weak chiral response, whereas fixing the orientation of pyrene and binaphthalene skeletons induces a more pronounced chiroptical effect.

**Fig. 2:**
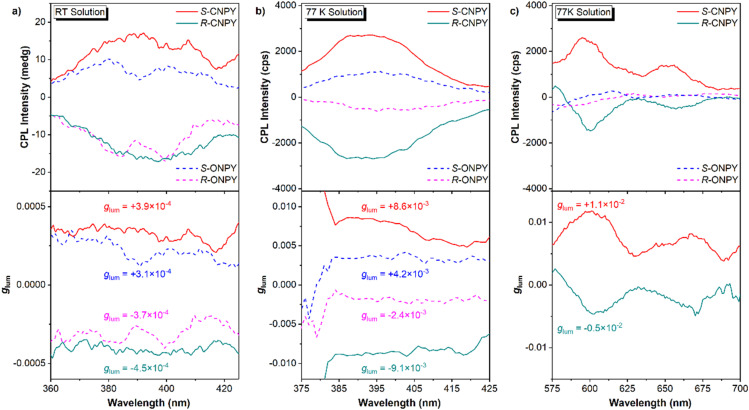
CPL spectra (top) and emissive dissymmetry factor *g*_lum_ (bottom) of *S*-CNPY, *R*-CNPY, *S*-ONPY, *R*-ONPY solutions in 2-MeTHF, (a) the fluorescence region (360–425 nm) at room temperature, (b) at 77 K, (c) the phosphorescence region (575–700 nm) at 77 K.

The CPL properties of enantiomers were then investigated to evaluate the effect of locked axial chirality on the luminescence. The CPL magnitude is assessed by the dissymmetry factors of *g*_lum_, which are defined as *g*_lum_ = 2× (*I*_L_ − *I*_R_)/(*I*_L_ + *I*_R_), where *I*_L_ and *I*_R_ indicate the intensity of Left-CPL and Right-CPL, respectively. Fig. 2a and b show the CPL spectra and *g*_lum_ in the fluorescence region at room temperature and 77 K, respectively. At room temperature, *R*-ONPY and *S*-ONPY show weak CPL signals with a quite low |*g*_lum_| of approximately 3 × 10^−4^. In contrast, *R*-CNPY and *S*-CNPY exhibit slightly higher CPL signals with a |*g*_lum_| of approximately 4 × 10^−4^. The |*g*_lum_| enhancement in CNPY enantiomers is probably attributed to structural rigidity induced by locked axial chirality and is conducive to stabilizing the molecular conformation at the excited states.

Lowering the temperature to 77 K, there is further enhance in the CPL intensity of both enantiomers, with CNPY maintaining a higher |*g*_lum_| (8.9 × 10^−3^) than ONPY (3.3 × 10^−3^). It is found that the |*g*_lum_| of CNPY at 77 K is about 20 times that measured at room temperature, whereas the corresponding ratio for ONPY is about 8. The progressive enhancement of |*g*_lum_| provides compelling evidence that environmental confinement and conformational restriction facilitate the amplification of CPL. Notably, CNPY enantiomers achieve detectable CPL in the red phosphorescence region (580–700 nm) at 77 K (Fig. 2c), with a |*g*_lum_| of about 10^−2^, while ONPY enantiomers show no measurable CPP. It is an impressive result, as it demonstrates that a locked axially chiral scaffold not only boosts the ISC channel to populate triplet excitons but also effectively transfers the chirality of the binaphthalene framework to the triplet states of the pyrene fragments, enabling CPP emission.

### Theoretical calculations

**Fig. 3:**
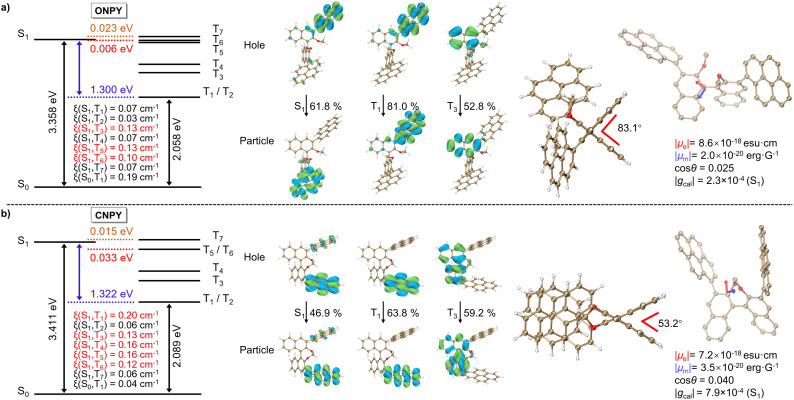
The calculated state-energy diagrams, the SOC coefficients (*ξ*), NTOs, optimized molecular structures, the simulated electric (*µ*_e_) and magnetic (*µ*_m_) transition dipole moments, and calculated *g*_cal_ for singlet states of (a) ONPY and (b) CNPY in their monomeric state, respectively.

To clarify the mechanism of locked axial chirality in regulating the photophysical (chiroptical) properties, we performed theoretical calculations. Fig. 3 (Left) presents the results of state-energy-level diagrams, spin–orbit coupling (SOC) coefficients (*ξ*), and natural transition orbitals (NTOs) of ONPY and CNPY, respectively. Compared to the PY (Fig. S4, SI), both ONPY and CNPY exhibit a smaller singlet–triplet energy gap (Δ*E*_ST_) between S_1_ and T_1_ (1.300 eV for ONPY, 1.322 eV for CNPY, and 1.651 eV for PY). Multiple accessible intersystem crossing pathways (7 channels for ONPY and CNPY *versus* 4 ones for PY), as well as higher spin–orbit coupling coefficients, are obtained, which should be responsible for the superior phosphorescence performance in ONPY and CNPY.

A detailed analysis of NTOs reveals that the axially chiral binaphthalene moiety is involved in the overall excited-state relaxation process, supporting intermediate triplet states (T_3_ and T_4_).^52,53^ It is consistent with experimental PL spectra in Fig. 1b. Furthermore, the axially chiral binaphthalene skeleton introduces the distorted excited-state configurations, which enable the circumferential migration of electrons between the π-orbitals of the twisted aromatic segments, employing the analogous El-Sayed's rule.^54^ The distorted configuration mixes singlet and triplet states to facilitate exciton exchange and spin-flip, and separates pyrene to prevent intramolecular excimer formation.^55,56^ Meanwhile, the pure (π,π*) characteristics in T_1_ of CNPY contribute to its persistent phosphorescence lifetime at 77 K. Overall, the binaphthalene scaffold mediates the phosphorescence properties.

Additionally, we selected the representative *S*-ONPY and *S*-CNPY for excited-state geometry optimization and CPL simulation. As shown in Fig. 3 (Right), the molecular configuration of *S*-ONPY is highly twisted, with a calculated dihedral angle of approximately 83.1° for the binaphthalene skeleton. In contrast, the dihedral angle of the locked binaphthalene skeleton in *S*-CNPY is 53.2°, significantly constraining the molecular geometry and providing superior structural rigidity. Based on these optimized structures, their electric transition dipole moments (|*µ*_e_|), magnetic transition dipole moments (|*µ*_m_|), and the intersection angles (*θ*) are calculated. Then, the dissymmetry factor *g*_cal_ are defined as *g*_cal_ = (4 cos*θ*|*µ*_e_‖*µ*_m_|)/(|*µ*_e_|^2^ + |*µ*_m_|^2^) ≈ (4 cos*θ*|*µ*_m_|)/|*µ*_e_|. As a result, *S*-CNPY has a higher |*g*_cal_| of 7.9 × 10^−4^, being approximately 3.4 times that of *S*-ONPY (2.3 × 10^−4^). It is in agreement with the results obtained in 2-MeTHF solutions, indicating the positive effect of locked axial chirality in CNPY.

### Circularly polarized room temperature phosphorescence

To evaluate the practical application potential, PY, ONPY, and CNPY were doped into a PVP matrix, and their photophysical properties were characterized at ambient conditions. PVP possesses abundant amide groups, which can restrict molecular motions *via* hydrogen bonding and van der Waals interactions with the dopant molecules. Meanwhile, its excellent solubility ensures uniform dispersion of the molecules and the dense film morphology. Also, we optimized the doping ratio and subject the resulting films to photo-thermal treatment before conducting photophysical measurements (Fig. S5, SI). The results demonstrate that the 1 : 500 doping ratio is the best condition. An excessively low doping ratio results in a significant decrease in afterglow brightness, while an overly high ratio induces excimer formation and subsequent phosphorescence quenching. Moreover, the phosphorescence of the films treated by photothermal treatment was significantly enhanced. FTIR analysis confirms the structural changes induced by photo-thermal treatment (Fig. S6, SI). The broad O–H/N–H vibrations at 3400 cm^−1^ are notably narrowed and weakened, indicating efficient removal of residual solvent and water. The strong C=O vibration at 1635 cm^−1^ is remarkably reduced, reflecting enhanced intermolecular hydrogen bonding between the dopant and PVP carbonyl groups. Additionally, the aromatic C=C (1421–1497 cm^−1^) and C–H out-of-plane bending (645 cm^−1^) peaks become sharper, demonstrating improved molecular dispersion and conformational rigidity of the dopant in the PVP matrix. These changes collectively establish a strong hydrogen-bonded network and suppress non-radiative vibrational relaxation, providing a structural foundation for enhanced phosphorescence and circularly polarized luminescence performance.

**Fig. 4:**
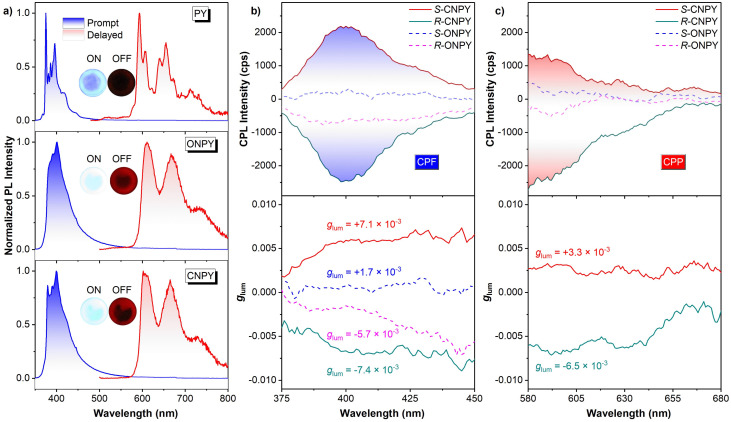
(a) Normalized prompt and delayed (1 ms) PL spectra in PVP films at room temperature for PY, ONPY, and CNPY, respectively. CPL spectra (top) and emissive dissymmetry factor *g*_lum_ (bottom) of PVP films, (b) the fluorescence region and (c) the phosphorescence region for *S*-ONPY, *S*-CNPY, *R*-ONPY, and *R*-CNPY, respectively, at room temperature.

Fig. 4a shows the normalized prompt and delayed PL spectra at room temperature for PY, ONPY, and CNPY PVP films. Similar to those observed in solutions at 77 K, these films also exhibit intense blue fluorescence at 360–500 nm and red phosphorescence at 570–800 nm, as illustrated by the inset pictures in Fig. 4a and the CIE coordinates in Fig. S8, SI. In detail, the fluorescence and phosphorescence emissions of both ONPY and CNPY are derived from the pyrene segments with the corresponding energy levels and vibrational features maintained. The lifetimes (Fig. S7, SI) and PL quantum yields (up to 69.6%, Fig. S9, SI) are also included, suggesting ultrabright PVP films with impressive red afterglow.

To deepen the understanding of excited-state relaxation processes, we calculated the photophysical parameters for PY, CNPY, and ONPY, as shown in Table S2, SI. The results indicate that the intersystem crossing rate (*k*_ISC_) of CNPY (1.50 × 10^7^ s^−1^) and ONPY (1.63 × 10^7^ s^−1^) is one to two orders of magnitude higher than that of pure pyrene (6.49 × 10^5^ s^−1^), which confirms that the binaphthalene axially chiral scaffold effectively boosts the ISC efficiency of pyrene. In addition, the phosphorescence rate constant (*k*_P_) of CNPY/ONPY is significantly lower than that of pyrene, while the non-radiative transition rate (*k*_NR_(T–S)) is basically same, which indicates that effective intermolecular interactions suppress the non-radiative decay from the triplet excited states of their pyrene moieties, and the extended π-conjugation in ONPY and CNPY endows them with a more prominent ^3^(π,π*) character in T_1_, thus yielding a longer phosphorescence lifetime in comparison with pyrene.

The CPL behaviours of the PVP films exhibit the same trend as observed in solutions. The CPL intensity exhibited a distinct enhancement with the molecular architecture transitioning from the open to the locked structure, with the |*g*_lum_| in CPF of CNPY enantiomers reaching 7.4 × 10^−3^, which is approximately twice that of ONPY enantiomers (Fig. 4b). Furthermore, a detectable CPP signal with a |*g*_lum_| of 6.5 × 10^−3^ was observed exclusively for CNPY enantiomers, whereas the CPP signal of ONPY was almost negligible (Fig. 4c). Furthermore, we also calculated another parameter, FM, which characterizes the overall performance of CPL. CNPY enantiomers have a higher FM value compared to that of ONPY enantiomers^57–59^ (Table S3, SI). Collectively, these phenomena corroborate the effectiveness of the strategy of locking axial chirality scaffolds, which suppresses conformational freedom and mediates transition dipole moments, facilitating CPL.

## Conclusions

In conclusion, this work demonstrates an effective strategy for achieving efficient circularly polarized luminescence and red circularly polarized phosphorescence in pyrene derivatives by manipulating a locked axially chiral scaffold. The structure-property relationship clearly demonstrates that introducing the chiral binaphthalene scaffold and locking its axial chirality are crucial for tailoring the luminescent properties of the pyrene moiety. Mediated by the transient triplet excited states of the binaphthalene unit, the overall intersystem crossing process is boosted. Combined with the enhanced molecular rigidity and optimized dipole moments, remarkably, the CNPY enantiomers doped into PVP achieve bright circularly polarized luminescence with a |*g*_lum_| of 7.4 × 10^−3^ and a *Φ*_PL_ of 65.7%, as well as distinct red circularly polarized phosphorescence with a |*g*_lum_| of ∼6.5 × 10^−3^ and *τ*_P_ of 381.9 ms. This work provides a new design principle for developing efficient CPL and red CPP materials.

## Author contributions

W. H. and C. W. performed all photophysical measurements, analysed data, synthesized materials, and performed theoretical calculations. M. W. assisted in characterizing molecular structure. Z. H. and Q. Z. designed and supervised the research. All authors discussed the results and commented on the manuscript.

## Conflicts of interest

There are no conflicts to declare.

## Supplementary Material

SC-OLF-D6SC01341D-s001

## Data Availability

The data supporting this article have been included as part of the supplementary information (SI). Supplementary information is available. See DOI: https://doi.org/10.1039/d6sc01341d.
