# Supplementary material for: Efficient red circularly-polarized phosphorescence from pyrene derivatives mediated by locked axial chirality scaffold
Source: Chem Sci. 2026 May 5;17(24):11830–7. doi: 10.1039/d6sc01341d (PMC13159748; doi:10.1039/d6sc01341d)
Supplement: SC-017-D6SC01341D-s001 [file SC-017-D6SC01341D-s001.pdf]

Supplementary Information (SI) for Chemical Science.
This journal is © The Royal Society of Chemistry 2026

Electronic Supplementary Material (ESI) for Chemical Science
The journal is © The Royal Society of Chemistry 2026

Supplemental Information

# Efficient Red Circularly-Polarized Phosphorescence from Pyrene Derivatives Mediated by Locked Axial Chirality Scaffold

Wenbin Huang[+], Chenlong Wei[+], Meng Wang, Qian Zhang, and Zikai He*

[+] These authors contributed equally to this work.

**Abstract:** As a classic polycyclic aromatic hydrocarbon luminophore, pyrene exhibits considerable potential for optoelectronic applications yet faces challenges in achieving chiral luminescence, especially the achievement of long-lived red circularly polarized phosphorescence. In this work, we report a strategy for manipulating the axial chirality of binaphthalene to enable pyrene to achieve efficient circularly polarized luminescence and red circularly polarized phosphorescence. It is revealed that the locked axial chiral binaphthalene not only boosts overall intersystem crossing via its transient triplet excited state but also amplifies circularly polarized phosphorescence through mediating structural rigidity and transition dipole moments. Bright circularly polarized luminescence with a high quantum yield of 65.7 % and distinct red circularly polarized phosphorescence with a dissymmetry factor of $6.5\times10^{-3}$ and a persistent lifetime of 381.9 ms, are obtained. The outlined structure-property relationship provides insights into the design principle for developing efficient circularly polarized luminescent materials.

**DOI:**

## General experimental details

All reactions were carried out under a nitrogen atmosphere. Starting materials and solvents were used without treatment except 2-methyltetrahydrofuran (2-MeTHF). $^1H$ spectra were carried out on QOne Instruments Quantum-I 400 M, and all NMR data were processed in MestReNova. All compounds were further purified by circulating liquid gel permeation chromatography with JAI LC-5060 equipped with JAIGEL-2.5HR and JAIGEL-2HR. UV-visible spectra were collected on a Shimadzu UV-2600. High-resolution mass spectroscopy was carried out on Waters ACQUITYUPLC H-Class/Xevo G2-XS QTof. All photophysical measurements, including steady-state or time-resolved spectra, decayed curves, and quantum yields, were carried out on an Edinburgh Instruments FLS1000 with various accessories. CPL measurement: Only solution samples at room temperature were used for comparison; the JASCO CPL-300 was used to collect data in transmission mode with a 180 ° light path. For other samples or situations, the Edinburgh Instruments FLS1000 CPL module is used to collect data in reflection mode with a 90 ° light path. Luminescence photos were taken with a Canon EOS 800D. High-performance liquid chromatography was carried out on Agilent 1260 LC equipped with DAICEL CHIRALPAK@IC. Infrared spectra were collected on Thermo Nicolet iS50 with ATR accessories.

The targeted molecules were purified multiple times by silica-gel column chromatography, gel-exclusion chromatography, and repeated recrystallization to ensure high purity for photophysical property investigations. The solvents, such as 2-methyltetrahydrofuran (2-MeTHF), were of high-performance liquid chromatography (HPLC) purity and were further distilled to remove stabilizers. Detailed synthetic routes were presented in the Supporting Information (Scheme S1). Poly(vinyl pyrrolidone) K 30 (PVP) (M. W. 40000) was used as the polymer matrix without further purification. Transparent doped films were prepared by mixing the guest solution with the PVP solution, then drop-coating the mixture onto a quartz plate and allowing natural evaporation. As-prepared films required further photothermal treatment to achieve a rigid, anhydrous state.

All ground-state geometry optimizations were performed using density functional theory (DFT) with the B3LYP functional and the 6-31G(d) basis set, as implemented in the Gaussian 16 package. Time-dependent density functional theory (TD‑DFT) at the CAM‑B3LYP/def2-SVP level was employed to obtain the singlet and triplet energies and electronic transition behaviors. To better match experimental conditions, solvent effects of tetrahydrofuran (THF) were incorporated using the polarizable continuum model (PCM). The electronic structure analysis, including frontier molecular orbital distributions and natural transition orbitals, was performed using Multiwfn[1, 2] and visualized with VMD. Spin–orbit coupling constants between singlet and triplet states were computed at the B3LYP/def2-SVP level with the ORCA package. Excited-state geometry optimizations were carried out using TD-DFT with the cam-B3LYP functional and the def2SVP basis set. Based on the optimized excited-state structures, the transition dipole moments from the excited state to the ground state were extracted via Multiwfn. All calculations incorporated the GD3BJ empirical dispersion correction to properly describe van der Waals interactions.

## Synthesis and characterization

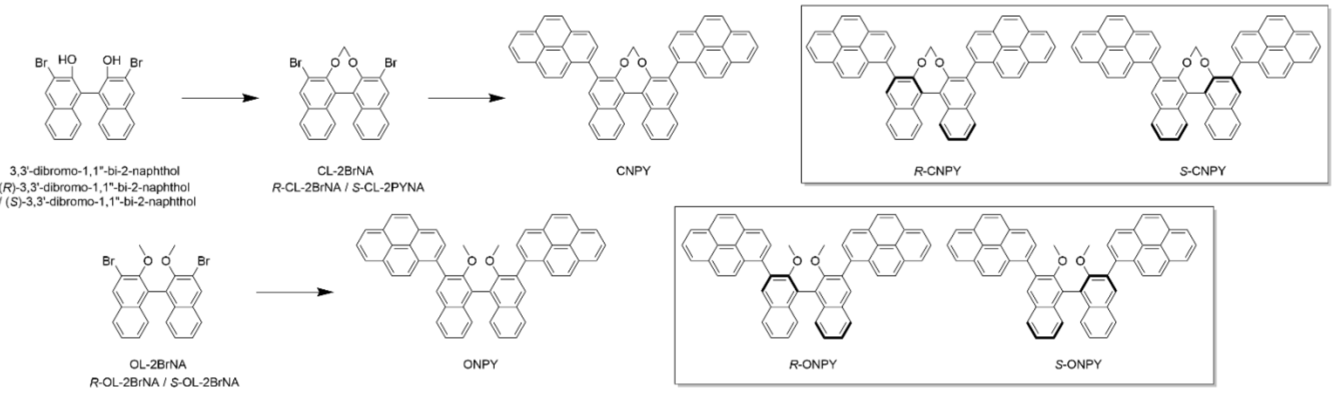


**Scheme S1.** Synthetic routes for compounds, together with labels for segments.

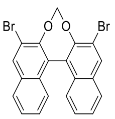

A 100 mL flask was charged with 3,3'-dibromo-1,1'-bi-2-naphthol (2.6515 g, 6.0 mmol), KOH (0.5037 g, 9.0 mmol), $CH_2I_2$ (1.8480 g, 6.9 mmol, 0.556 mL) under a nitrogen atmosphere. 60 mL anhydrous DMF was injected into the flask, and the resulting mixture was maintained at 333 K for 12 hours. Subsequently, the solvent was removed from the resulting solution by vacuum distillation. Afterwards, the residue was purified by silica gel column chromatography using dichloromethane/petroleum ether (20:80, v/v) as the eluent. Yield: 42.1 % (1146.60 mg). The chiral isomers (*R*-CL-2BrNA and *S*-CL-2BrNA) were obtained by a similar process in which the chiral raw material replaces the achiral raw material. Yield: 40.9 % (1113.92 mg) for *R*-CL-2BrNA and 39.0 % (1062 .17 mg) for *S*-CL-2BrNA.

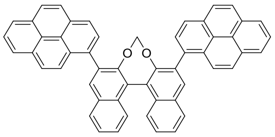

A 100 mL flask was charged with CL-2BrNA (0.8478 g, 2.0 mmol), 1-Pyrenylboronic acid (1.0828 g, 4.4 mmol), Tetrakis(triphenylphosphine)palladium (0.1387 g, 0.12 mmol), and potassium carbonate (1.2162 g, 8.8 mmol) under a nitrogen atmosphere. Degassed solvent, including water (5 mL), ethyl alcohol (5 mL), and toluene (40 mL), was injected into the flask, and the resulting mixture was maintained at 383 K for 6 hours. Subsequently, it was extracted with dichloromethane and rotary evaporated to yield the residue, which was purified by silica-gel column chromatography using dichloromethane/petroleum ether (33:67 v/v) as the eluent, followed by recrystallization for further purification. Yield: 70.2 % (980.31 mg). The chiral isomers (*R*-CNPY and *S*-CNPY) were obtained by a similar process in which the chiral raw material replaces the achiral raw material. Yield: 65.5 % (914.67 mg) for *R*-CNPY and 67.8 % (946.79 mg) for *S*-CNPY. $^1$H NMR (400 MHz, Methylene Chloride-$d_2$) δ 8.25 – 7.74 (m, 24H), 7.64 – 7.46 (m, 4H), 4.85 – 4.81 (m, 2H). $^{13}$C NMR (101 MHz, Methylene Chloride-$d_2$) δ 154.19, 133.82, 133.65, 133.48, 133.33, 133.19, 131.60, 131.47, 130.60, 130.11, 129.89, 128.53, 128.39, 127.38, 126.63, 125.84, 125.69, 125.25, 125.04, 124.90, 124.75, 124.64, 124.33, 124.17, 123.95, 123.76, 59.81. HRMS (ESI) for CNPY Calcd. 699.2324, Found 699.2323 $[C_{53}H_{30}O_2+H]^+$.

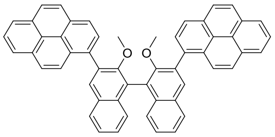

A 100 mL flask was charged with OL-2BrNA (0.9399 g, 2.0 mmol), 1-Pyrenylboronic acid (1.0828 g, 4.4 mmol), Tetrakis(triphenylphosphine)palladium (0.1387 g, 0.12 mmol), and potassium carbonate (1.2162 g, 8.8 mmol) under a nitrogen atmosphere. Degassed solvent, including water (5 mL), ethyl alcohol (5 mL), and toluene (40 mL), was injected into the flask, and the resulting mixture was maintained at 383 K for 6 hours. Subsequently, it was extracted with dichloromethane and rotary evaporated to yield a residue, which was purified by silica-gel column chromatography using dichloromethane/petroleum ether (33:67 v/v) as the eluent, followed by recrystallization for further purification. Yield: 75.1 % (1072.81 mg). The chiral isomers (*R*-ONPY and *S*-ONPY) were obtained by a similar process in which the chiral raw material replaces the achiral raw material. Yield: 69.9 % (998.53 mg) for *R*-ONPY and 76.5 % (1092.81 mg) for *S*-ONPY. $^1$H NMR (400 MHz, Chloroform-*d*) δ 8.24 – 7.82 (m, 28H), 7.64 – 7.45 (m, 4H), 4.85 – 4.80 (m, 2H). $^{13}$C NMR (101 MHz, Chloroform-*d*) δ 150.01, 134.27, 133.73, 133.40, 133.08, 132.49, 132.21, 132.09, 131.88, 131.39, 131.08, 130.89, 129.81, 128.70, 128.49, 127.70, 127.42, 127.06, 126.40, 126.12, 126.00, 125.75, 125.28, 125.09, 124.73, 124.45, 102.99. HRMS (ESI) for ONPY Calcd. 715.2637, Found 715.2640 $[C_{54}H_{34}O_2+H]^+$.

Treated film: 1 mg target molecule was dissolved in 1 mL dichloromethane to obtain a clarified guest solution by 1 minute of ultrasound treatment. Similarly, 500 mg Poly(vinyl pyrrolidone) K 30 (PVP) was dissolved in 9 mL dichloromethane to obtain a clarified host solution. Then, the 1 mL guest and 9 mL host solutions were mixed and ultrasonicated for 5 minutes to prepare a mixed solution with a mass

ratio of 1:500 (guest: host). Drop the mixed solution onto a 1 cm-diameter circular quartz plate and allow it to dry at room temperature. The as-prepared film was heated at 393 K for 30 minutes and then excited with 365 nm ultraviolet light at room temperature for 10 minutes to obtain a treated PVP film doped with the guest.

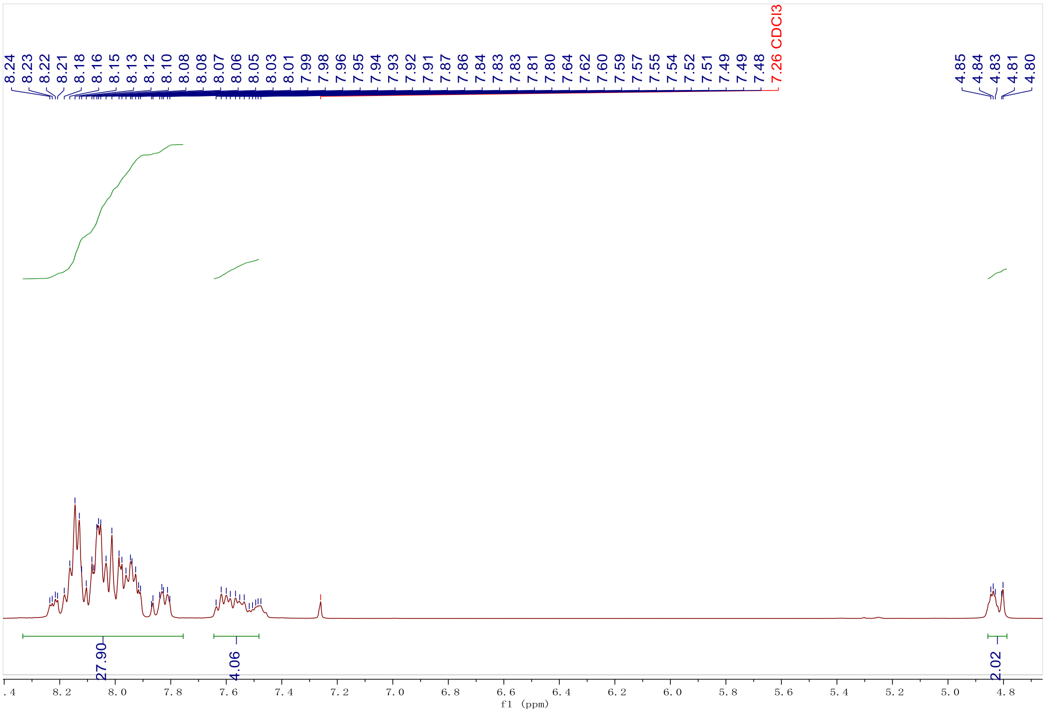


**Chart S1.** $^{1}H$ NMR spectrum of ONPY in Chloroform-*d*.

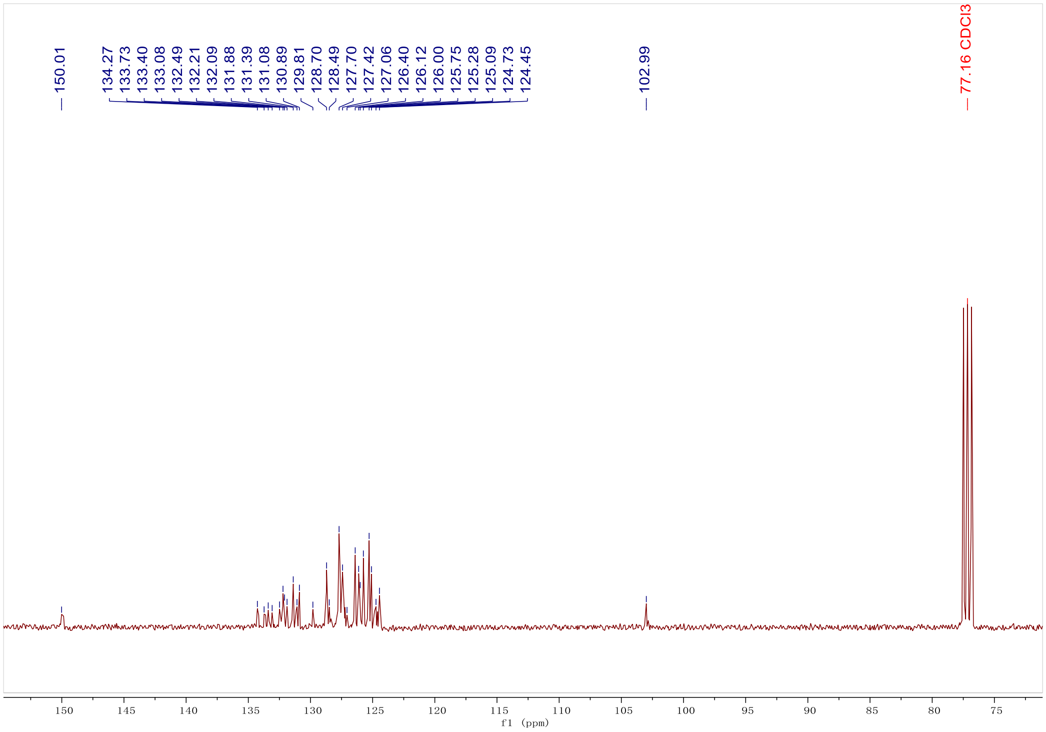


**Chart S2.** $^{13}C$ NMR spectrum of ONPY in Chloroform-*d*.

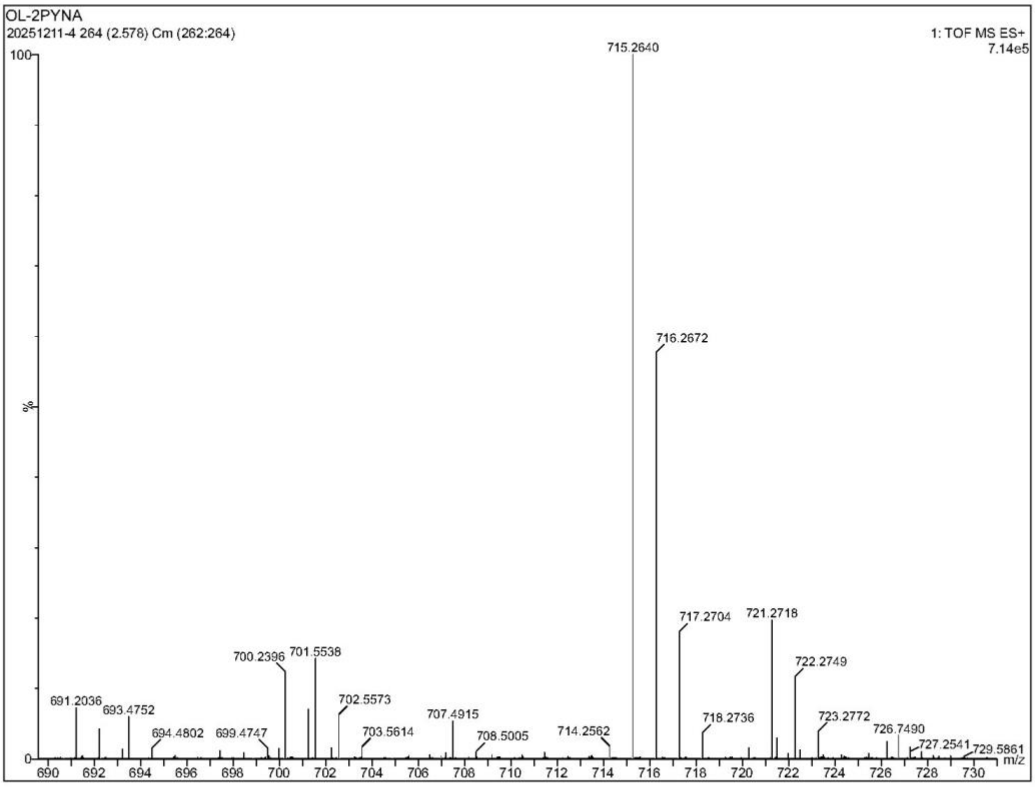


**Chart S3.** High-resolution mass spectrum of ONPY.

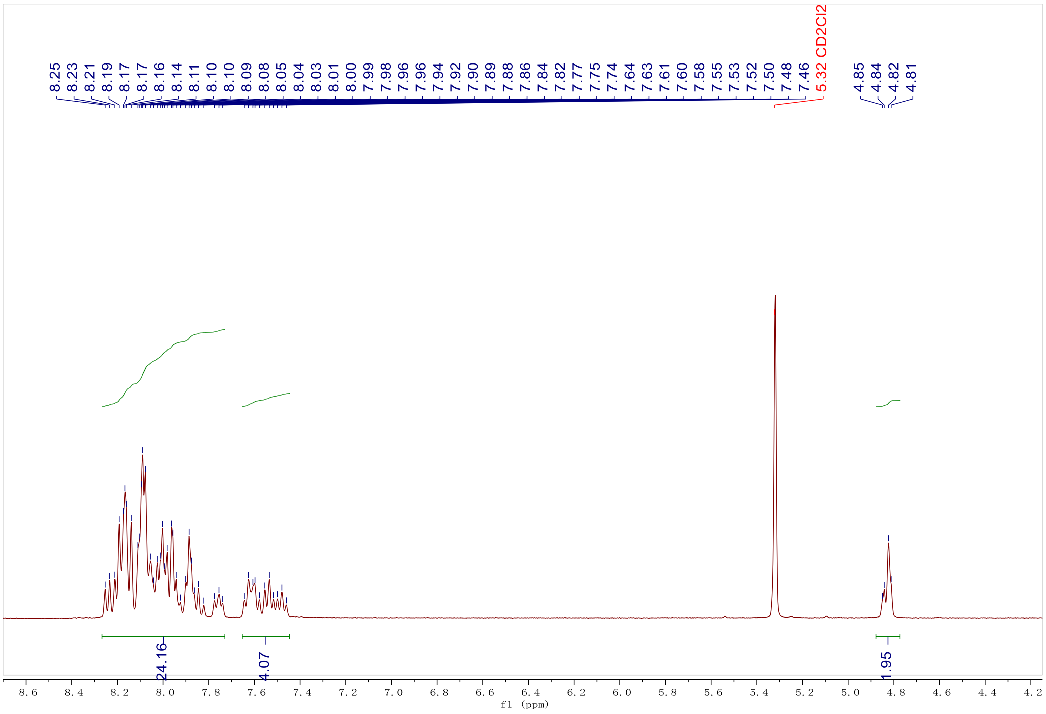


**Chart S4.** $^{1}$H NMR spectrum of CNPY in Methylene Chloride-$d_2$.

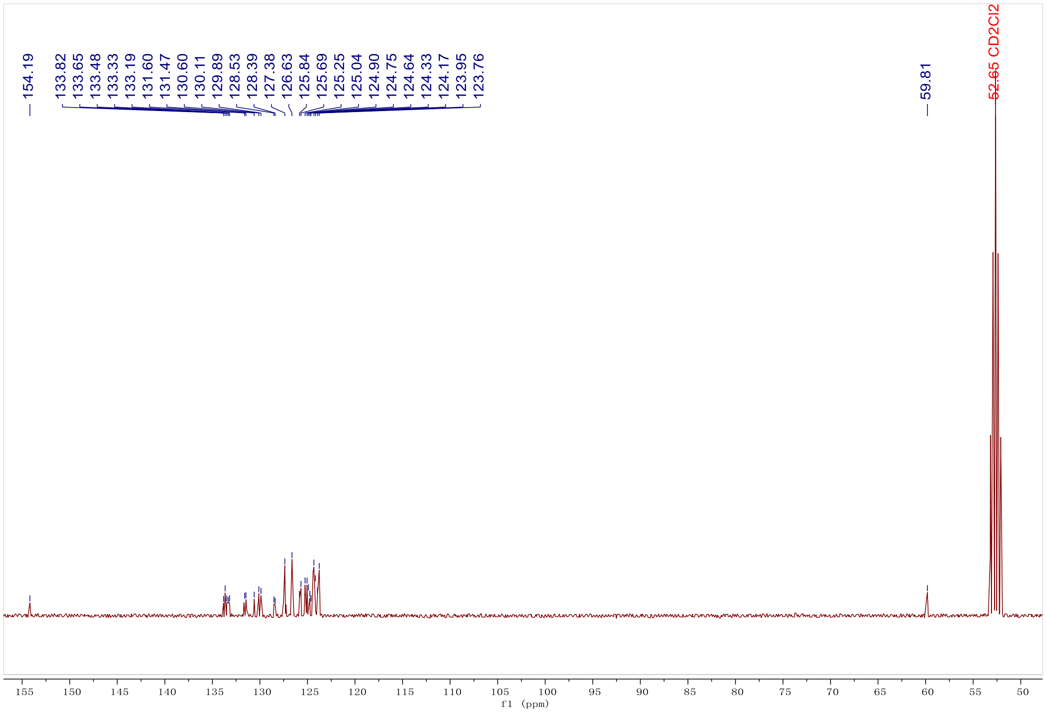


**Chart S5.** $^{13}C$ NMR spectrum of CNPY in Methylene Chloride-$d_2$.

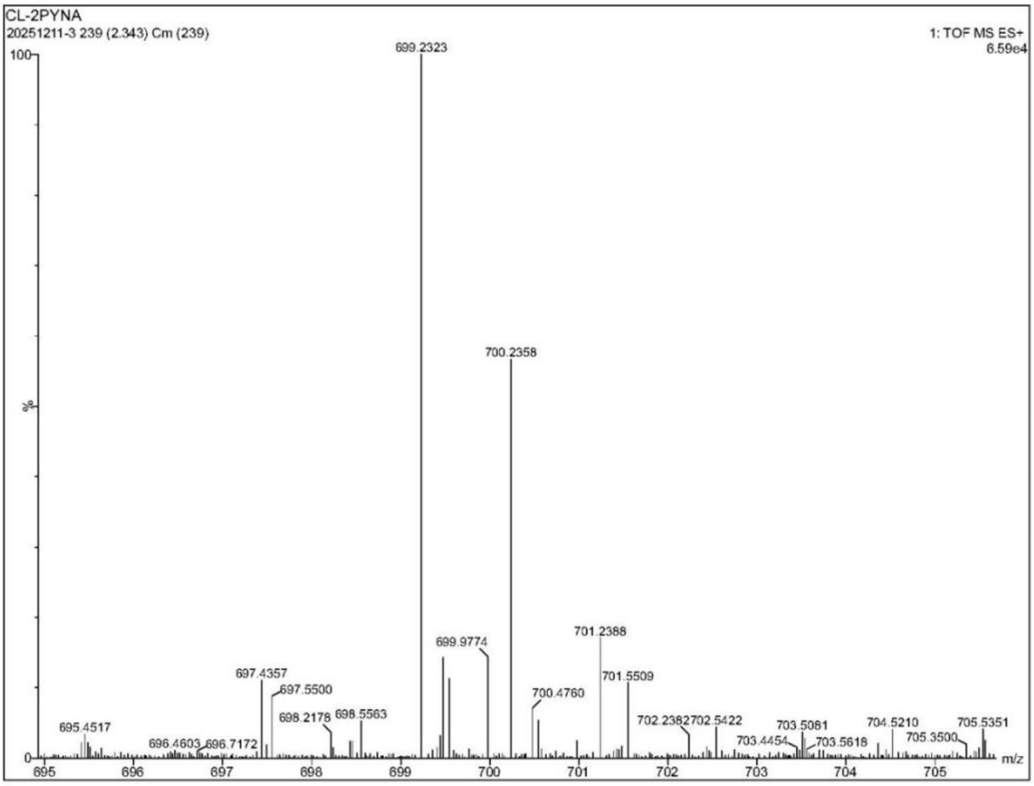


**Chart S6.** High-resolution mass spectrum of CNPY.

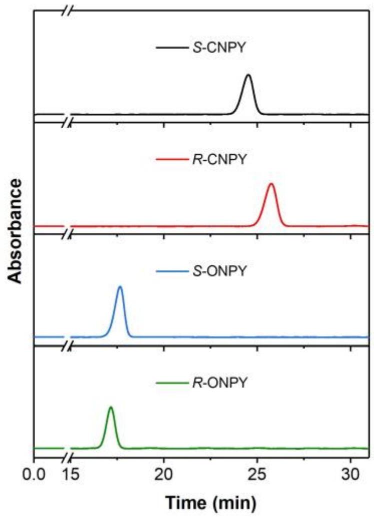


**Chart S7.** High performance liquid chromatography of *S*-CNPY, *R*-CNPY, *S*-ONPY, and *R*-ONPY (Temperature: 30℃; Mobile phase: 90% acetonitrile and 10% water; Flow rate: 1.0 mL/min; Column: DAICEL CHIRALPAK IC 4.6 mm × 250 mm, 5 μm; Detection signal: 330 nm).

## Supplementary photophysical data

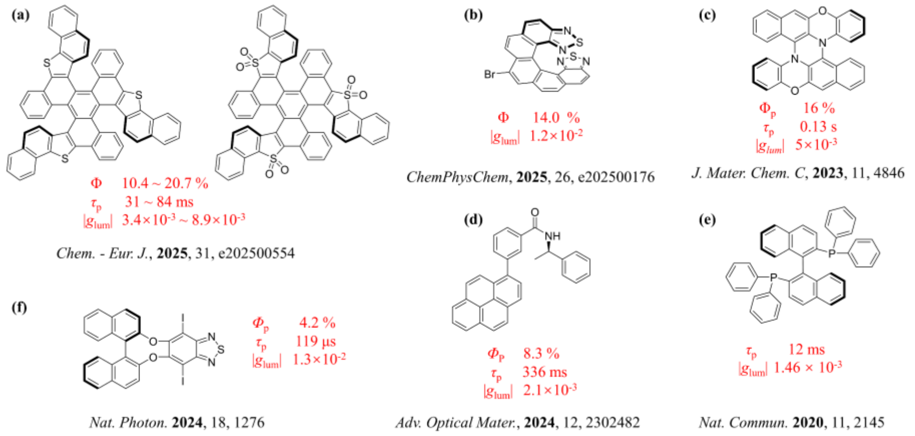


**Figure S1.** The summaries of the performance in recently reported pure organic red circularly polarized phosphorescent small molecules[3-8].

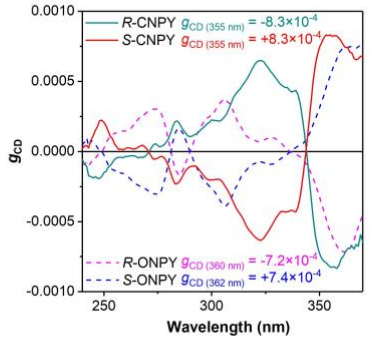


**Figure S2.** $g_{lum}$ in solution at RT for *R*-CNPY, *S*-CNPY, *R*-ONPY, *S*-ONPY, respectively.

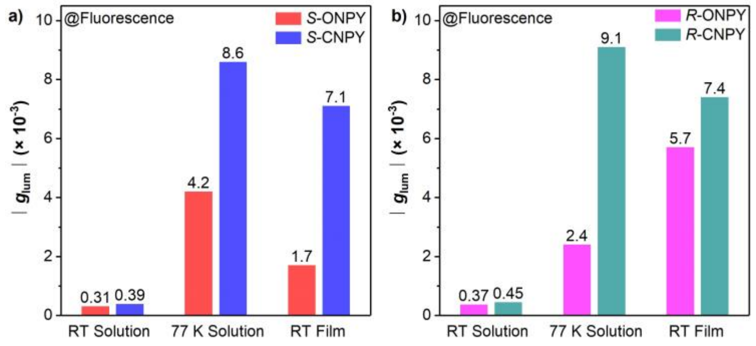


**Figure S3.** $|g_{lum}|$ of circularly polarized fluorescence for *S*-ONPY, *S*-CNPY, *R*-ONPY, and *R*-CNPY under different environmental conditions.

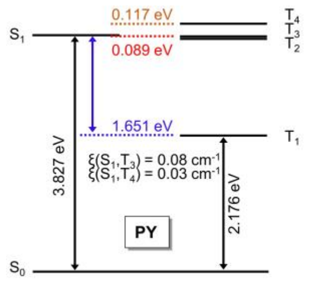


**Figure S4.** The state-energy-level diagrams and the SOC coefficients (ξ) for Pyrene in the monomeric state.

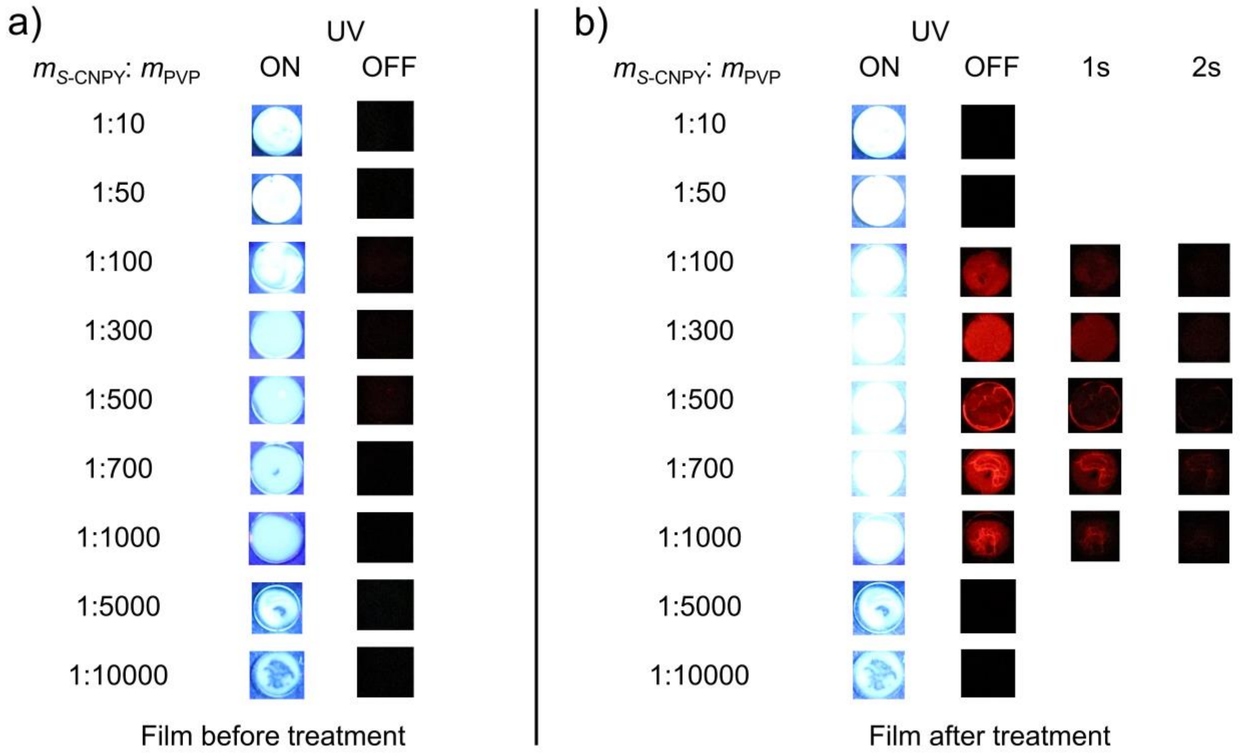


**Figure S5.** Photographs of (a) untreated and (b) treated films with different concentrations of doping *S*-CNPY at a 365 nm UV excitation under ambient conditions.

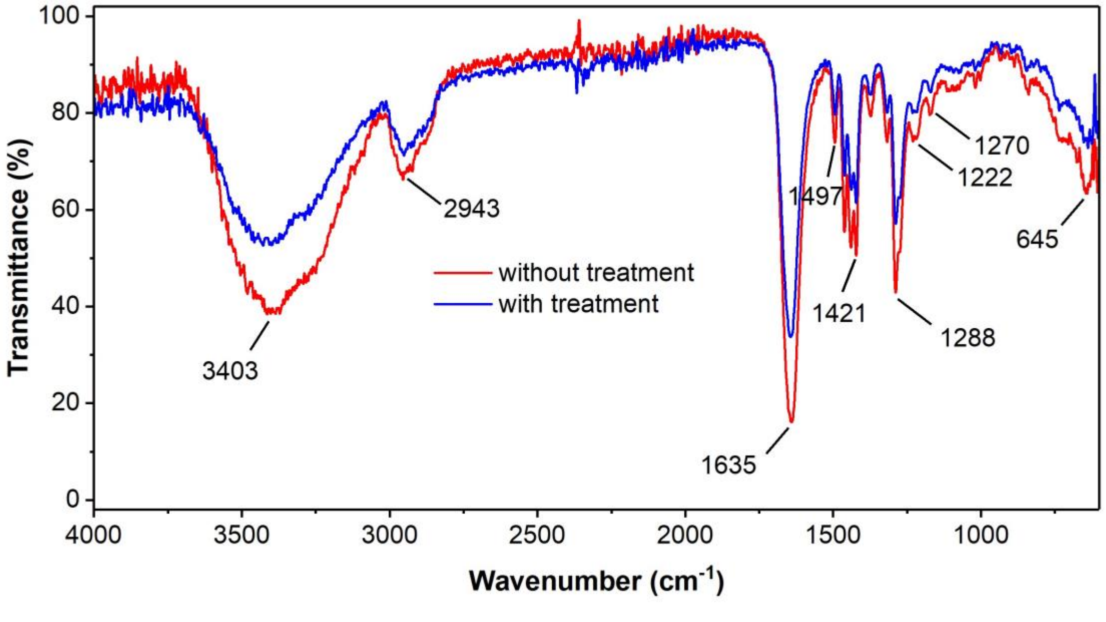


**Figure S6.** Infrared spectroscopy of PVP films doped with S-CNPY before and after photo-thermal treatment.

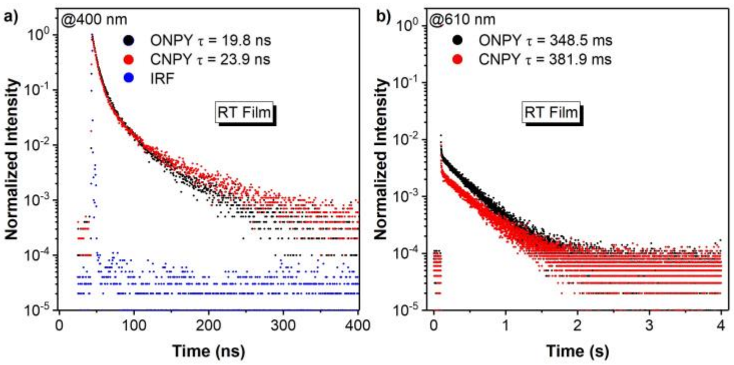


**Figure S7.** Time-resolved (a) fluorescence and (b) phosphorescence decay curves and their respective lifetimes for ONPY and CNPY films under ambient conditions.

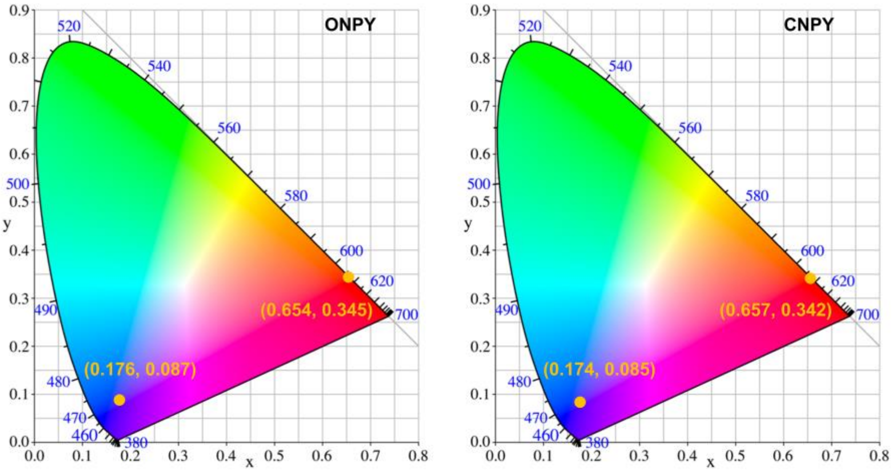


**Figure S8.** CIE coordinates of fluorescence and phosphorescence for ONPY and CNPY films, respectively.

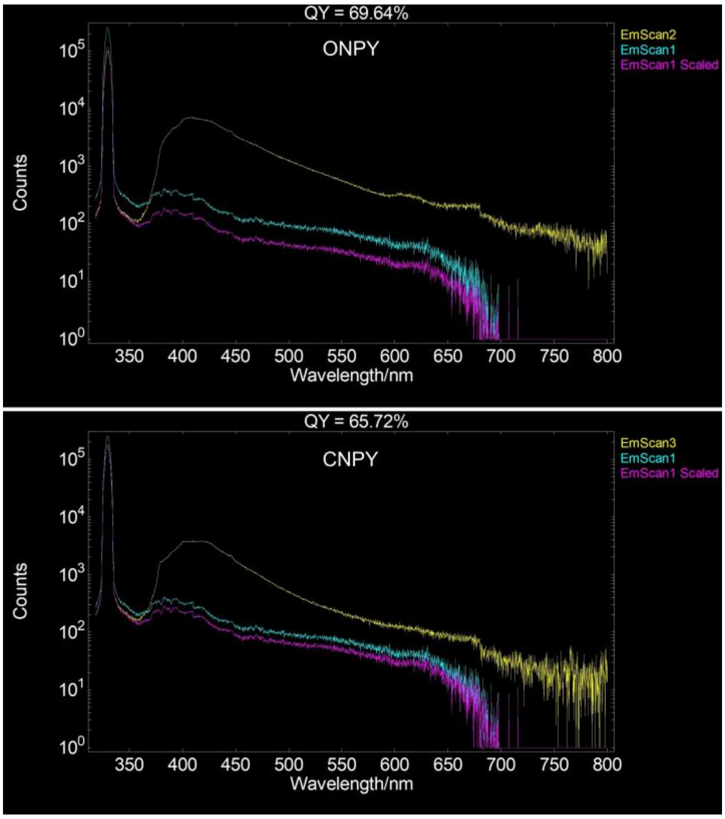


**Figure S9.** Quantum yield of ONPY and CNPY films under ambient conditions.

**Table S1.** Photophysical properties of compounds measured at room temperature and 77 K.

| Name | State | Emission lifetime | Quantum yield |
|---|---|---|---|
| ONPY | Solution<br>(In 2-MeTHF, $1\times10^{-5}$ M) | 400 nm (16.8 ns, 77 K)<br>600 nm (484.4 ms, 77 K) | - |
| | Treated film<br>(In PVP, 1:500) | 400 nm (19.8 ns, RT)<br>610 nm (348.5 ms, RT) | 69.64 % |
| CNPY | Solution<br>(In 2-MeTHF, $1\times10^{-5}$ M) | 400 nm (34.6 ns, 77 K)<br>600 nm (525.0 ms, 77 K) | - |
| | Treated film<br>(In PVP, 1:500) | 400 nm (23.9 ns, RT)<br>610 nm (381.9 ms, RT) | 65.72 % |

**Table S2.** Calculated photophysical parameters for PY, ONPY and CNPY films at room temperature.

| Sample | $k_{P}$[$s^{-1}$] | $k_{NR(T-S)}$[$s^{-1}$] | $k_{ISC}$[$s^{-1}$] |
|---|---|---|---|
| ONPY | 0.17 | 2.7 | $1.63\times10^{7}$ |
| CNPY | 0.12 | 2.5 | $1.50\times10^{7}$ |
| PY | 0.60 | 2.1 | $6.49\times10^{5}$ |

**Table S3.** Calculated FM of CPL for *S*-ONPY and *S*-CNPY films at room temperature[9].

| Sample | *S*-ONPY | *S*-CNPY |
|---|---|---|
| FM (CPF) | $1.15\times10^{-3}$ | $4.53\times10^{-3}$ |
| FM (CPP) | - | $6.50\times10^{-5}$ |

## Theoretical calculation details

**Table S4.** Calculated SOC of molecules based on the optimized geometry of $S_1$ and $T_n$ at B3LYP/def2-SVP.

| | PY | ONPY | CNPY |
|---|---|---|---|
| ($cm^{-1}$) | $S_1$ | $S_1$ | $S_1$ |
| $T_1$ | 0.00 | 0.07 | 0.20 |
| $T_2$ | 0.00 | 0.03 | 0.06 |
| $T_3$ | 0.08 | 0.13 | 0.13 |
| $T_4$ | 0.03 | 0.07 | 0.16 |
| $T_5$ | 0.00 | 0.13 | 0.16 |
| $T_6$ | 0.00 | 0.10 | 0.12 |
| $T_7$ | 0.00 | 0.07 | 0.06 |

**Table S5.** Calculated energy levels of molecules based on the optimized geometry of $S_1$, $S_2$, and $T_n$.

| (eV) | PY | ONPY | CNPY |
|---|---|---|---|
| $S_1$ | 3.827 | 3.344 | 3.411 |
| $S_2$ | 4.082 | 3.462 | 3.412 |
| $T_1$ | 2.176 | 2.058 | 2.089 |
| $T_2$ | 3.728 | 2.060 | 2.090 |
| $T_3$ | 3.738 | 2.631 | 2.487 |
| $T_4$ | 3.944 | 2.648 | 2.698 |
| $T_5$ | 4.398 | 3.338 | 3.378 |
| $T_6$ | 4.554 | 3.352 | 3.381 |
| $T_7$ | 4.589 | 3.367 | 3.426 |
| $T_8$ | 5.104 | 3.413 | 3.434 |
| $T_9$ | 5.232 | 3.464 | 3.480 |
| $T_{10}$ | 5.337 | 3.472 | 3.487 |

**Table S6.** Transition moments of *S*-ONPY

| Ground to excited state transition electric dipole moments (Au): | | | | | |
|---|---|---|---|---|---|
| state | X | Y | Z | Dip. S. | Osc. |
| 1 | 3.4002 | -1.0191 | -0.3147 | 12.6988 | 1.1497 |
| 2 | -0.8952 | -1.979 | 0.7755 | 5.3193 | 0.485 |

| | | | | | |
|---|---|---|---|---|---|
| 3 | -0.0717 | 0.0299 | 0.0438 | 0.008 | 0.0008 |
| 4 | 0.046 | 0.0696 | -0.0081 | 0.007 | 0.0007 |
| 5 | -0.968 | 0.158 | -0.5236 | 1.2362 | 0.1303 |
| 6 | -0.4871 | -0.0064 | -0.3236 | 0.342 | 0.0364 |
| 7 | 0.7211 | -0.3427 | 0.9358 | 1.5132 | 0.1622 |
| 8 | 0.4339 | -0.6366 | -0.3804 | 0.7382 | 0.0801 |
| 9 | -0.7375 | -0.2416 | -0.0862 | 0.6097 | 0.0697 |
| 10 | -0.1347 | 0.0392 | 0.0692 | 0.0245 | 0.0028 |

Ground to excited state transition magnetic dipole moments (Au):

| state | X | Y | Z |
|---|---|---|---|
| 1 | 0.0726 | 1.5924 | 2.3484 |
| 2 | 0.811 | -0.6917 | 1.7467 |
| 3 | 0.0306 | 0.163 | 0.0074 |
| 4 | 0.0024 | -0.0232 | 0.1874 |
| 5 | 0.2985 | 0.0659 | -0.9381 |
| 6 | 0.4763 | -0.1782 | -0.0252 |
| 7 | -0.5473 | -0.0525 | 0.5577 |
| 8 | 0.4991 | 0.0473 | 0.1686 |
| 9 | -0.2485 | -1.0776 | -0.6615 |
| 10 | 1.461 | 0.3975 | 2.3407 |

Rotatory Strengths (R) in cgs (10**-40 erg-esu-cm/Gauss)

| state | XX | YY | ZZ | R(velocity) | E-M Angle |
|---|---|---|---|---|---|
| 1 | 1724.909 | -792.1435 | 549.0615 | 493.9423 | 77.87 |
| 2 | -1478.513 | 3.4257 | 78.736 | -465.4505 | 115.05 |
| 3 | -0.537 | 0.0836 | -0.568 | -0.3405 | 93.44 |
| 4 | 1.6482 | -0.0533 | -0.3384 | 0.4188 | 86.09 |
| 5 | -119.5961 | 59.0434 | -105.9184 | -55.4903 | 101.88 |
| 6 | 25.6515 | 124.4991 | 17.8795 | 56.01 | 40.25 |
| 7 | 36.2438 | 55.1614 | -186.5008 | -31.6985 | 97.76 |
| 8 | -329.0897 | 157.6529 | 81.0774 | -30.1198 | 106.03 |
| 9 | -92.1848 | -26.758 | -246.9935 | -121.9788 | 119.88 |
| 10 | -66.7195 | 25.3659 | 66.0895 | 8.2453 | 85.36 |

Rotatory Strengths (R) in cgs (10**-40 erg-esu-cm/Gauss)

| state | XX | YY | ZZ | R(length) |
|---|---|---|---|---|
| 1 | -174.5429 | 1147.6152 | 522.6156 | 498.5626 |
| 2 | 513.4331 | -967.9929 | -957.8734 | -470.8111 |
| 3 | 1.554 | -3.4443 | -0.2299 | -0.7067 |
| 4 | -0.0766 | 1.1401 | 1.067 | 0.7102 |
| 5 | 204.3677 | -7.357 | -347.3672 | -50.1188 |
| 6 | 164.079 | -0.8012 | -5.7586 | 52.5064 |
| 7 | 279.0841 | -12.7142 | -369.1017 | -34.2439 |
| 8 | -153.1167 | 21.3057 | 45.3484 | -28.8209 |
| 9 | -129.6271 | -184.0973 | -40.3031 | -118.0092 |
| 1 | -174.5429 | 1147.6152 | 522.6156 | 498.5626 |

**Table S7.** Summary of chiroptical properties of *S*-ONPY.

| state | $\lvert\mu_e\rvert$ ($10^{-20}$ esu-cm) | $\lvert\mu_m\rvert$ ($10^{-20}$ erg/Gauss) | $\cos\theta$ (E-M Angle) | $g$ ($10^{-4}$ $4R/D$) | $E_{ex}$ (nm) |
|---|---|---|---|---|---|
| 1 | 857.12 | 1.96 | -0.025 | -2.30 | 388.55 |
| 2 | 24.24 | 0.09 | -0.461 | -71.20 | 332.26 |
| 3 | 642.05 | 1.54 | -0.051 | -4.86 | 318.31 |
| 4 | 67.98 | 0.17 | -0.237 | -24.03 | 311.80 |
| 5 | 36.57 | 0.36 | 0.625 | 246.41 | 307.73 |

Dissymmetry factor (g) was calculated as follows: $g = 4R/D$, where R is the rotatory strength defined by the inner product of transition electric and magnetic dipole moments ($R = |\mu_e|\cdot|\mu_m|\cdot\cos\theta$). D is the dipole strength defined by the sum of squares of transition electric and magnetic dipole moments ($D = |\mu_e|^2 + |\mu_m|^2$). $E_{ex}$ is the excitation energy calculated by the TD-DFT method at the cam-B3LYP/def2-SVP level of theory.

**Table S8.** Calculated excited states of *S*-ONPY.

| | | | | | | |
|---|---|---|---|---|---|---|
| Excited State | 1: | Singlet-A | 3.1910 eV | 388.55 nm | f=0.8878 | <S**2>=0.000 |
| 187 ->188 | | 0.69188 | | | | |
| Excited State | 2: | Singlet-A | 3.7316 eV | 332.26 nm | f=0.0008 | <S**2>=0.000 |
| 180 ->188 | | 0.19176 | | | | |
| 182 ->188 | | 0.10471 | | | | |
| 183 ->188 | | 0.39888 | | | | |
| 184 ->188 | | 0.1337 | | | | |
| 187 ->193 | | 0.46771 | | | | |
| 187 ->194 | | 0.10837 | | | | |
| Excited State | 3: | Singlet-A | 3.8951 eV | 318.31 nm | f=0.6081 | <S**2>=0.000 |
| 182 ->192 | | -0.15257 | | | | |

| | | | | | | |
|---|---|---|---|---|---|---|
| 186 ->189 | | 0.66691 | | | | |
| Excited State | 4: | Singlet-A | 3.9764 eV | 311.80 nm | f=0.0070 | <S**2>=0.000 |
| 181 ->189 | | -0.15546 | | | | |
| 182 ->189 | | 0.40771 | | | | |
| 183 ->189 | | -0.12472 | | | | |
| 186 ->190 | | 0.15492 | | | | |
| 186 ->192 | | 0.46859 | | | | |
| Excited State | 5: | Singlet-A | 4.0291 eV | 307.73 nm | f=0.0020 | <S**2>=0.000 |
| 180 ->191 | | 0.14892 | | | | |
| 183 ->188 | | -0.14396 | | | | |
| 183 ->191 | | -0.14119 | | | | |
| 184 ->188 | | 0.38466 | | | | |
| 184 ->194 | | 0.13533 | | | | |
| 185 ->188 | | -0.20662 | | | | |
| 187 ->190 | | -0.13968 | | | | |
| 187 ->191 | | -0.32034 | | | | |
| 187 ->193 | | -0.11486 | | | | |

**Table S9.** Transition moments of *S*-CNPY.

| Ground to excited state transition electric dipole moments (Au): | | | | | |
|---|---|---|---|---|---|
| state | X | Y | Z | Dip. S. | Osc. |
| 1 | -0.9841 | 0.019 | 1.4639 | 3.1117 | 0.2896 |
| 2 | 0.0094 | 3.2053 | -0.0085 | 10.2739 | 0.9642 |
| 3 | 0.1788 | -0.0199 | -0.0058 | 0.0324 | 0.0031 |
| 4 | -0.0098 | -0.3755 | 0.0003 | 0.1411 | 0.0136 |
| 5 | 1.9604 | -0.0023 | -0.0939 | 3.852 | 0.3868 |
| 6 | 0.0016 | 0.2393 | -0.0008 | 0.0573 | 0.0061 |
| 7 | 0.0475 | -0.0002 | -0.2316 | 0.0559 | 0.0061 |
| 8 | 0.001 | -0.095 | 0.0003 | 0.009 | 0.001 |
| 9 | -0.0054 | -0.2734 | -0.0094 | 0.0749 | 0.0085 |
| 10 | 0.0924 | -0.0138 | 0.1696 | 0.0375 | 0.0043 |

| Ground to excited state transition magnetic dipole moments (Au): | | | |
|---|---|---|---|
| state | X | Y | Z |
| 1 | 1.3303 | 0.008 | -3.9893 |

| | | | |
|---|---|---|---|
| 2 | -0.0054 | 1.9463 | 0.0196 |
| 3 | 0.0403 | 0.0002 | 0.3108 |
| 4 | -0.0019 | 0.0069 | -0.0165 |
| 5 | 0.2005 | -0.0006 | 1.5845 |
| 6 | -0.0012 | -0.4462 | 0.0013 |
| 7 | -0.3413 | 0.0011 | -0.1125 |
| 8 | -0.0009 | -0.0721 | 0.0003 |
| 9 | 0.0876 | -0.427 | -0.0513 |
| 10 | -1.6505 | -0.0221 | 0.976 |

Rotatory Strengths (R) in cgs (10**-40 erg-esu-cm/Gauss)

| state | XX | YY | ZZ | R(velocity) | E-M Angle |
|---|---|---|---|---|---|
| 1 | 3788.6794 | 1207.8348 | 21.1826 | 1672.5656 | 15.43 |
| 2 | -4211.1486 | 0.0369 | -137.8926 | -1449.6681 | 179.2 |
| 3 | -14.8522 | -7.1854 | 2.7951 | -6.4142 | 112.04 |
| 4 | 4.9607 | -0.0196 | -2.9774 | 0.6546 | 68 |
| 5 | -13.1611 | -551.7818 | 384.5003 | -60.1475 | 94.52 |
| 6 | 37.4332 | 0.0013 | 31.0661 | 22.8335 | 90 |
| 7 | -19.3578 | 4.2543 | 8.8208 | -2.0942 | 96.31 |
| 8 | 12.9166 | -0.0003 | -17.7481 | -1.6106 | 90 |
| 9 | -18.39 | 0.1371 | -67.1297 | -28.4609 | 166.39 |
| 10 | -111.5606 | 41.5943 | 65.4425 | -1.5079 | 90.9 |

Rotatory Strengths (R) in cgs (10**-40 erg-esu-cm/Gauss)

| state | XX | YY | ZZ | R(length) |
|---|---|---|---|---|
| 1 | 925.7941 | -0.1074 | 4129.8188 | 1685.1685 |
| 2 | 0.0358 | -4411.5403 | 0.118 | -1470.4622 |
| 3 | -5.1001 | 0.0034 | 1.267 | -1.2766 |
| 4 | -0.0135 | 1.8393 | 0.0036 | 0.6098 |
| 5 | -277.9788 | -0.0009 | 105.2049 | -57.5916 |
| 6 | 0.0013 | 75.5164 | 0.0007 | 25.1728 |
| 7 | 11.4701 | 0.0002 | -18.4345 | -2.3214 |
| 8 | 0.0006 | -4.8418 | -0.0001 | -1.6137 |
| 9 | 0.3316 | -82.5496 | -0.34 | -27.5193 |
| 10 | 925.7941 | -0.1074 | 4129.8188 | 1685.1685 |

**Table S10.** Summary of chiroptical properties of *S*-CNPY.

| state | $\lvert\mu_e\rvert$ ($10^{-20}$ esu-cm) | $\lvert\mu_m\rvert$ ($10^{-20}$ erg/Gauss) | cos$\theta$ (E-M Angle) | $g$ ($10^{-4}$ 4$R$/$D$) | $E_{ex}$ (nm) |
|---|---|---|---|---|---|
| 1 | 716.84 | 3.54 | 0.040 | 7.93 | 375.32 |
| 2 | 15.83 | 0.13 | -0.880 | -0.03 | 329.79 |
| 3 | 459.32 | 1.92 | 0.217 | 0.00 | 314.10 |
| 4 | 277.13 | 1.23 | -0.136 | 0.00 | 210.30 |
| 5 | 352.24 | 1.51 | -0.005 | 0.00 | 309.24 |

Dissymmetry factor (g) was calculated as follows: $g = 4R/D$, where R is the rotatory strength defined by the inner product of transition electric and magnetic dipole moments ($R = |\mu_e|\cdot|\mu_m|\cdot\cos\theta$). D is the dipole strength defined by the sum of squares of transition electric and magnetic dipole moments ($D = |\mu_e|^2 + |\mu_m|^2$). $E_{ex}$ is the excitation energy calculated by the TD-DFT method at the cam-B3LYP/def2SVP level of theory.

**Table S11.** Calculated excited states of *S*-CNPY.

| | | | | | | |
|---|---|---|---|---|---|---|
| Excited State | 1: | Singlet-A | 3.3034 eV | 375.32 nm | f=0.6428 | <S**2>=0.000 |
| 182 ->183 | | 0.68615 | | | | |
| Excited State | 2: | Singlet-A | 3.7595 eV | 329.79 nm | f=0.0004 | <S**2>=0.000 |
| 176 ->183 | | -0.22352 | | | | |
| 178 ->183 | | -0.39468 | | | | |
| 182 ->186 | | 0.11556 | | | | |
| 182 ->188 | | -0.4798 | | | | |
| Excited State | 3: | Singlet-A | 3.9473 eV | 314.10 nm | f=0.3154 | <S**2>=0.000 |
| 179 ->183 | | 0.13559 | | | | |
| 180 ->183 | | -0.44929 | | | | |
| 180 ->185 | | 0.29652 | | | | |
| 180 ->186 | | -0.112 | | | | |
| 181 ->184 | | 0.29303 | | | | |
| Excited State | 4: | Singlet-A | 3.9957 eV | 310.30 nm | f=0.1162 | <S**2>=0.000 |
| 177 ->184 | | 0.34466 | | | | |
| 177 ->187 | | 0.11177 | | | | |
| 180 ->183 | | 0.16856 | | | | |
| 181 ->184 | | 0.38761 | | | | |
| 181 ->187 | | -0.36192 | | | | |
| Excited State | 5: | Singlet-A | 4.0093 eV | 309.24 nm | f=0.1884 | <S**2>=0.000 |
| 177 ->184 | | -0.30277 | | | | |
| 177 ->187 | | 0.13929 | | | | |
| 180 ->183 | | 0.15359 | | | | |
| 181 ->184 | | 0.43734 | | | | |
| 181 ->185 | | -0.13084 | | | | |

| | |
|---|---|
| 181 ->187 | 0.32667 |

## XYZ coordinates of optimized molecular structure

PY

| | | | |
|---|---|---|---|
| C | -3.36661300 | -0.00679400 | -0.10182400 |
| C | -2.69756500 | 1.15439600 | -0.06733600 |
| C | -1.35327100 | 1.16326900 | -0.02661000 |
| C | -0.67415400 | -0.00136100 | -0.02041500 |
| C | -1.34770200 | -1.16871900 | -0.05513900 |
| C | -2.69202500 | -1.16527200 | -0.09570300 |
| C | -0.67554300 | 2.32332800 | 0.00813600 |
| C | 0.66444200 | 2.32603200 | 0.04868500 |
| C | 1.34770200 | 1.16872000 | 0.05511900 |
| C | 0.67415600 | 0.00136000 | 0.02038000 |
| C | 1.35327200 | -1.16326900 | 0.02657600 |
| C | 0.67554600 | -2.32332700 | -0.00821700 |
| C | -0.66444000 | -2.32603200 | -0.04875200 |
| C | 2.69202500 | 1.16527200 | 0.09575300 |
| C | 3.36661000 | 0.00679300 | 0.10190500 |
| C | 2.69756500 | -1.15439600 | 0.06735400 |
| H | -4.46913500 | -0.00901900 | -0.13514800 |
| H | -3.27321600 | 2.09571800 | -0.07314700 |
| H | -3.26317700 | -2.10890800 | -0.12456200 |
| H | -1.20624300 | 3.29090100 | 0.00398800 |
| H | 1.19051600 | 3.29573800 | 0.07651800 |
| H | 1.20624600 | -3.29090000 | -0.00409900 |
| H | -1.19051300 | -3.29573700 | -0.07660100 |
| H | 3.26317300 | 2.10890700 | 0.12465600 |
| H | 4.46913000 | 0.00901800 | 0.13530300 |
| H | 3.27321600 | -2.09571800 | 0.07317900 |

ONPY

| | | | |
|---|---|---|---|
| C | -2.07131800 | 5.17459000 | -2.49821200 |
| C | -0.80217100 | 5.19895700 | -1.87220000 |
| C | -0.34127800 | 4.10100600 | -1.18132300 |
| C | -1.12868100 | 2.92423200 | -1.08020600 |
| C | -2.41273800 | 2.90010800 | -1.71147500 |
| C | -2.85665000 | 4.04784100 | -2.41948900 |
| C | -0.68682100 | 1.77106700 | -0.36308900 |
| C | -1.49524400 | 0.65311600 | -0.30291300 |
| C | -2.77617100 | 0.61112700 | -0.93236100 |
| C | -3.20501300 | 1.72978200 | -1.61861300 |
| C | 0.62488500 | 1.77322900 | 0.34499600 |
| C | 0.73710700 | 2.33249200 | 1.65418700 |
| C | 2.00447000 | 2.29709900 | 2.31799800 |
| C | 3.11500300 | 1.72674400 | 1.65007900 |

| | | | |
|---|---|---|---|
| C | 3.01138200 | 1.19792700 | 0.37862000 |
| C | 1.73745000 | 1.22955300 | -0.26872500 |
| C | -0.36818300 | 2.91580800 | 2.33042000 |
| C | -0.22609000 | 3.43308000 | 3.59873700 |
| C | 1.02774700 | 3.39742200 | 4.25423700 |
| C | 2.11754300 | 2.84270400 | 3.62384800 |
| O | -1.03323300 | -0.46757700 | 0.35735700 |
| C | -1.57066900 | -0.64647000 | 1.67049600 |
| O | 1.61000000 | 0.74859600 | -1.55011600 |
| C | 1.60397400 | -0.68082300 | -1.65782500 |
| C | -3.59966300 | -0.62432000 | -0.88106400 |
| C | 4.20468400 | 0.66321300 | -0.32623800 |
| C | -3.06391900 | -1.81624700 | -1.38812200 |
| C | -3.80222000 | -2.99091100 | -1.41335900 |
| C | -5.11539600 | -3.02528300 | -0.92143800 |
| C | -5.67652700 | -1.83280200 | -0.37496400 |
| C | -4.91117800 | -0.62402100 | -0.34707600 |
| C | -7.00464500 | -1.85507000 | 0.14661000 |
| C | -7.57205500 | -0.67642800 | 0.71616900 |
| C | -6.76914500 | 0.51107400 | 0.76398700 |
| C | -5.50471300 | 0.53717800 | 0.26122100 |
| C | -5.90883100 | -4.22069400 | -0.94694100 |
| C | -7.17921600 | -4.23700400 | -0.45954600 |
| C | -7.77258100 | -3.05871100 | 0.10601700 |
| C | -9.07819600 | -3.05392500 | 0.62084300 |
| C | -9.62431100 | -1.89627200 | 1.17040400 |
| C | -8.88064800 | -0.71937000 | 1.22052700 |
| C | 4.98671700 | -0.38424100 | 0.21869000 |
| C | 6.16094800 | -0.81318000 | -0.47754800 |
| C | 6.52622100 | -0.20160300 | -1.71373800 |
| C | 5.70679300 | 0.81009500 | -2.23571100 |
| C | 4.57143800 | 1.22773600 | -1.55594200 |
| C | 4.63258000 | -1.07141800 | 1.43230800 |
| C | 5.40468200 | -2.07260900 | 1.93604100 |
| C | 6.60771000 | -2.49606500 | 1.27997900 |
| C | 6.97239200 | -1.85834200 | 0.05696100 |
| C | 8.15182400 | -2.27628900 | -0.63184000 |
| C | 8.49397200 | -1.63173500 | -1.86789500 |
| C | 7.71483800 | -0.64340300 | -2.38542800 |
| C | 7.42121100 | -3.51946000 | 1.78949600 |
| C | 8.57358800 | -3.91528000 | 1.11425200 |
| C | 8.93600500 | -3.30283200 | -0.08325300 |
| H | -2.42153000 | 6.04753500 | -3.04154000 |
| H | -0.18701400 | 6.09169000 | -1.94049800 |
| H | 0.63290500 | 4.11998400 | -0.70488900 |
| H | -3.83205700 | 4.01788200 | -2.89862700 |
| H | -4.17034300 | 1.70912400 | -2.11717000 |
| H | 4.08124800 | 1.72655800 | 2.14727800 |
| H | -1.32888500 | 2.95114300 | 1.82805700 |
| H | -1.08192100 | 3.87600300 | 4.10024000 |

| | | | |
|---|---|---|---|
| H | 1.12611200 | 3.81060400 | 5.25390400 |
| H | 3.08586700 | 2.81196300 | 4.11713600 |
| H | -1.28399600 | 0.18861000 | 2.32008600 |
| H | -2.66271600 | -0.73537500 | 1.64121300 |
| H | -1.14097800 | -1.57464000 | 2.05212900 |
| H | 0.78942100 | -1.10214200 | -1.06084600 |
| H | 1.44978800 | -0.89985600 | -2.71633200 |
| H | 2.56099900 | -1.10622000 | -1.33534500 |
| H | -2.05223400 | -1.80588900 | -1.77866300 |
| H | -3.36682900 | -3.89734900 | -1.82581700 |
| H | -7.19261200 | 1.40106200 | 1.22267200 |
| H | -4.91684100 | 1.44551600 | 0.32242800 |
| H | -5.46812000 | -5.12012300 | -1.36921300 |
| H | -7.76957900 | -5.14922600 | -0.48715800 |
| H | -9.66161400 | -3.97035400 | 0.58692700 |
| H | -10.63641600 | -1.91207300 | 1.56459300 |
| H | -9.30910700 | 0.18050700 | 1.65413400 |
| H | 5.97505700 | 1.27466500 | -3.18095500 |
| H | 3.94993800 | 2.01215800 | -1.97329500 |
| H | 3.72014300 | -0.78392400 | 1.94133100 |
| H | 5.11161800 | -2.57961200 | 2.85193900 |
| H | 9.39281300 | -1.95511200 | -2.38652200 |
| H | 7.98096200 | -0.16556100 | -3.32477400 |
| H | 7.13943300 | -4.00339300 | 2.72105300 |
| H | 9.19268200 | -4.70880300 | 1.52299400 |
| H | 9.83431000 | -3.61771700 | -0.60793200 |

CNPY

| | | | |
|---|---|---|---|
| C | -3.56021100 | 2.72974700 | 0.21390400 |
| C | -2.89103000 | 3.86294500 | 0.74065700 |
| C | -1.45936300 | 3.91505500 | 0.70853100 |
| C | -0.74074500 | 2.84189600 | 0.08537500 |
| C | -1.44737100 | 1.72772100 | -0.34125400 |
| C | -2.86505500 | 1.65297500 | -0.29146400 |
| C | -3.61349600 | 4.93213100 | 1.33343900 |
| C | -2.95827600 | 5.99756600 | 1.90424600 |
| C | -1.54397700 | 6.02853200 | 1.91587000 |
| C | -0.81471200 | 5.01574000 | 1.33564200 |
| C | 0.73498800 | 2.84323000 | -0.08474200 |
| C | 1.45168300 | 3.91741300 | -0.70835700 |
| C | 2.88343300 | 3.86763800 | -0.74081500 |
| C | 3.55463200 | 2.73570800 | -0.21389100 |
| C | 2.86137800 | 1.65800200 | 0.29209300 |
| C | 1.44361300 | 1.73045600 | 0.34217200 |
| C | 0.80507900 | 5.01683000 | -1.33568200 |
| C | 1.53254400 | 6.03062800 | -1.91641100 |
| C | 2.94689500 | 6.00199500 | -1.90510500 |
| C | 3.60399800 | 4.93782300 | -1.33411100 |

| | | | |
|---|---|---|---|
| O | -0.76043500 | 0.66960900 | -0.90369800 |
| O | 0.75843200 | 0.67125900 | 0.90479300 |
| C | 0.00008600 | -0.11194700 | 0.00040100 |
| C | -3.56527400 | 0.43334500 | -0.78186900 |
| C | 3.56361900 | 0.43955600 | 0.78259400 |
| C | 4.11998900 | 0.42798100 | 2.06433800 |
| C | 4.76196900 | -0.70055100 | 2.56411000 |
| C | 4.86479000 | -1.86697300 | 1.79379900 |
| C | 4.31328700 | -1.87353700 | 0.47724300 |
| C | 3.66625900 | -0.70798900 | -0.03406800 |
| C | 5.50570300 | -3.05543300 | 2.28275400 |
| C | 5.59271100 | -4.17754700 | 1.51827500 |
| C | 5.04963100 | -4.21720100 | 0.18920600 |
| C | 4.41012700 | -3.04874200 | -0.32517000 |
| C | 3.86709700 | -3.06270100 | -1.64479700 |
| C | 3.24174600 | -1.86985100 | -2.13972000 |
| C | 3.14948600 | -0.74645000 | -1.37562200 |
| C | 5.12706200 | -5.36295800 | -0.61680700 |
| C | 4.59147900 | -5.36795300 | -1.90325700 |
| C | 3.96885800 | -4.23172500 | -2.41450300 |
| C | -4.12293300 | 0.42135400 | -2.06305400 |
| C | -4.76303500 | -0.70823300 | -2.56285500 |
| C | -4.86264500 | -1.87531500 | -1.79312800 |
| C | -4.30993000 | -1.88144500 | -0.47707400 |
| C | -3.66485100 | -0.71484100 | 0.03427800 |
| C | -4.40364300 | -3.05726900 | 0.32480400 |
| C | -3.85952400 | -3.07076900 | 1.64398700 |
| C | -3.23635400 | -1.87683800 | 2.13905100 |
| C | -3.14702900 | -0.75287000 | 1.37543400 |
| C | -5.50147100 | -3.06486100 | -2.28217200 |
| C | -5.58542300 | -4.18757700 | -1.51823900 |
| C | -5.04112200 | -4.22679800 | -0.18965600 |
| C | -5.11546400 | -5.37314300 | 0.61581200 |
| C | -4.57881500 | -5.37768400 | 1.90182100 |
| C | -3.95819100 | -4.24040800 | 2.41316300 |
| H | -4.64569500 | 2.69398600 | 0.25049700 |
| H | -4.69939500 | 4.88212800 | 1.33889100 |
| H | -3.52131600 | 6.80691400 | 2.35992900 |
| H | -1.02810000 | 6.85636900 | 2.39392800 |
| H | 0.26757000 | 5.04626900 | 1.36180400 |
| H | 4.64016300 | 2.70171900 | -0.25078700 |
| H | -0.27725500 | 5.04557200 | -1.36161400 |
| H | 1.01519400 | 6.85746000 | -2.39461700 |
| H | 3.50849600 | 6.81212300 | -2.36117700 |
| H | 4.68997700 | 4.88960400 | -1.33980100 |
| H | -0.64203800 | -0.72827800 | 0.63422700 |
| H | 0.64385400 | -0.72622900 | -0.63376000 |
| H | 4.03244700 | 1.31868200 | 2.67929700 |
| H | 5.18313600 | -0.68732300 | 3.56595700 |
| H | 5.92163600 | -3.03845300 | 3.28686500 |

| | | | |
|---|---|---|---|
| H | 6.07896400 | -5.07048000 | 1.90259800 |
| H | 2.84380600 | -1.87749000 | -3.15118000 |
| H | 2.68587700 | 0.14858800 | -1.77696500 |
| H | 5.61255700 | -6.25261100 | -0.22412000 |
| H | 4.66087300 | -6.26476500 | -2.51224300 |
| H | 3.55444200 | -4.24016500 | -3.41920600 |
| H | -4.03781900 | 1.31257700 | -2.67759800 |
| H | -5.18519200 | -0.69531400 | -3.56429000 |
| H | -2.83769600 | -1.88411700 | 3.15023100 |
| H | -2.68506600 | 0.14296300 | 1.77690500 |
| H | -5.91832700 | -3.04820800 | -3.28590600 |
| H | -6.07011400 | -5.08133100 | -1.90262800 |
| H | -5.59940500 | -6.26361000 | 0.22305500 |
| H | -4.64581400 | -6.27496200 | 2.51038800 |
| H | -3.54295400 | -4.24849300 | 3.41752900 |

The calculated natural transition orbitals of the frontier excited singlet and triplet states

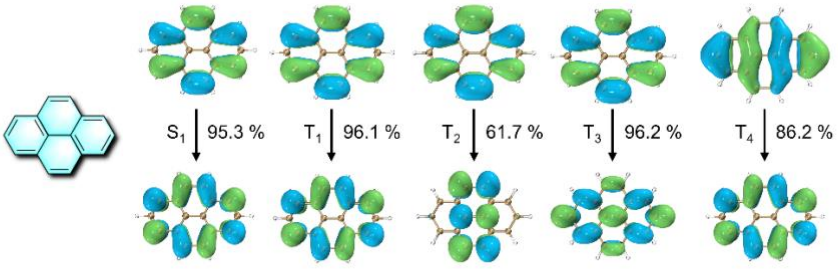


**Figure S10.** NTOs for Pyrene.

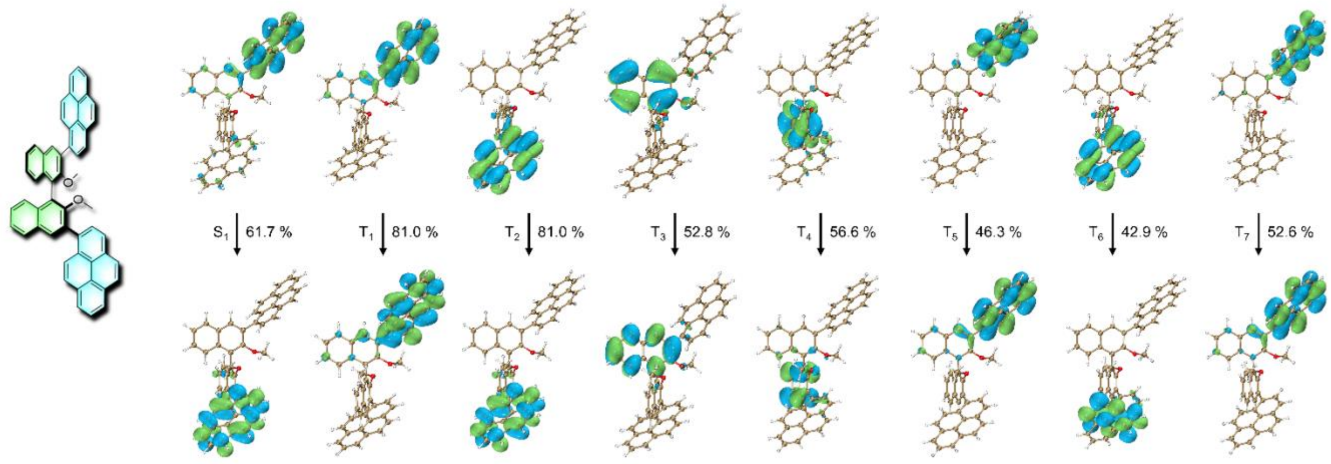


**Figure S11.** NTOs for *S*-ONPY.

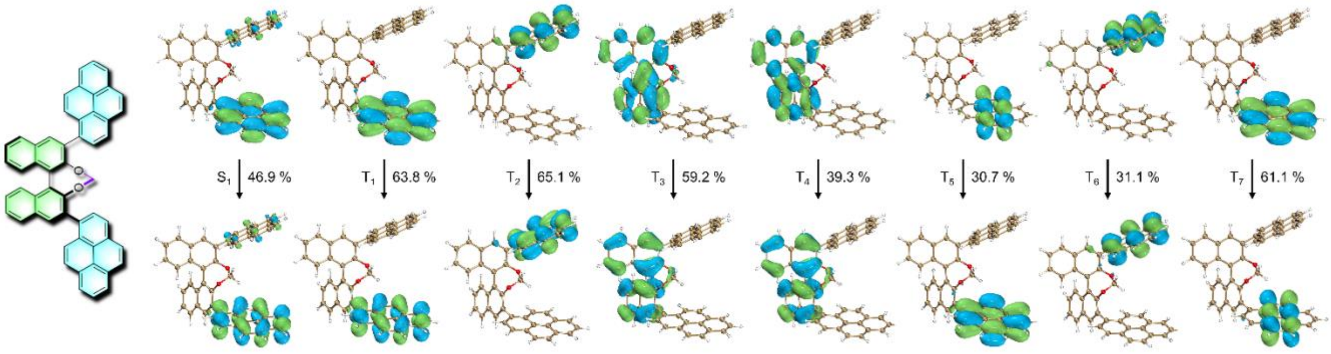


**Figure S12.** NTOs for *S*-CNPY.

## Author Contributions

W.H. and C.W. performed all photophysical measurements, analyzed data, synthesized materials, and performed theoretical calculations. M.W. assisted in characterizing molecular structure. Z.H. and Q.Z. designed and supervised the research. All authors discussed the results and commented on the manuscript.

# Analysis of decay rate constants

As discussed in the manuscript, the importance of the $k_{ISC}$ value, its details, and the calculation equations are presented here.

As we know,

$$\Phi_{ISC} = \frac{k_{ISC}}{k_{ISC} + k_F + k_{IC}}$$

$$\Phi_F = \frac{k_F}{k_{ISC} + k_F + k_{IC}}$$

considering the larger proportion of phosphorescence emission relative to fluorescence, we assume that

$$\Phi_{ISC} + \Phi_F = 1. \ (k_{IC} = 0 \text{ s}^{-1})$$

Therefore,

$$\Phi_{ISC} = \frac{k_{ISC}}{k_{ISC} + k_F} = k_{ISC} \cdot \tau_F$$

$$\Phi_F = \frac{k_F}{k_{ISC} + k_F}$$

After conversion,

$$k_{ISC} = \frac{1 - \Phi_F}{\tau_F}$$

Because

$$\Phi_P = \Phi_{ISC} \frac{k_p}{k_P + k_{nr}}$$

$$k_P = \frac{1 - k_{nr}\tau_P}{\tau_P}$$

Therefore,

$$k_P = \frac{\Phi_P}{(1 - \Phi_F)\tau_P}$$

$$k_{nr} = \frac{1}{\tau_P} - \frac{\Phi_P}{(1 - \Phi_F)\tau_P}$$

where $\tau_P$ and $\tau_F$ represent the phosphorescence and fluorescence lifetime, the $k_P$, $k_F$, $k_{ISC}$, and $k_{nr}$ represent the decay rate constants for phosphorescence, fluorescence, intersystem crossing, and nonradiative transition from $T_1$, $\Phi_P$, $\Phi_F$, and $\Phi_{ISC}$, indicate the efficiency of phosphorescence, fluorescence, and intersystem crossing, respectively.

## References


1. T. Lu and F. Chen, Multiwfn: A multifunctional wavefunction analyzer, *J. Comput. Chem.*, 2012, **33**, 580-592.
2. T. Lu, A comprehensive electron wavefunction analysis toolbox for chemists, Multiwfn, *J. Chem. Phys.*, 2024, **161**, 082503.
3. S. Qiu, W. Li, S. Zhang, W. Xu, J. Tang, W. Tian and H. Wang, C3-symmetric Triple Thia/Sulfone[6]Helicene with Dual Emissions, *Chem. - Eur. J.*, 2025, **31**, e202500554.
4. D. Kundu, R. Chowdhury, N. Del Rio, M. Cordier, N. Vanthuyne, R. H. Friend, M. Srebro-Hooper and J. Crassous, Bromo-Heptahelicene-Bis-Thiadiazole: Photophysics, Chiroptics, and Excited-State Dynamics, *ChemPhysChem*, 2025, **26**, e202500176.
5. S. Tanaka, D. Sakamaki, N. Haruta, T. Sato, M. Gon, K. Tanaka and H. Fujiwara, A double heterohelicene composed of two benzo[b]phenothiazine exhibiting intense room-temperature circularly polarized phosphorescence, *J. Mater. Chem. C*, 2023, **11**, 4846-4854.
6. Y. Jiang, C. Zhang, R. Wang, Y. Lei, W. Dai, M. Liu, H. Wu, Y. Tao and X. Huang, Chiral-Guest Induced Multicolor-Tunable Circularly Polarized Room Temperature Phosphorescence, *Adv. Opt. Mater.*, 2024, **12**, 2302482.
7. X. Wu, C.-Y. Huang, D.-G. Chen, D. Liu, C. Wu, K.-J. Chou, B. Zhang, Y. Wang, Y. Liu, E. Y. Li, W. Zhu and P.-T. Chou, Exploiting racemism enhanced organic room-temperature phosphorescence to demonstrate Wallach's rule in the lighting chiral chromophores, *Nat. Commun.*, 2020, **11**, 2145.
8. D. Liu, W.-J. Wang, P. Alam, Z. Yang, K. Wu, L. Zhu, Y. Xiong, S. Chang, Y. Liu, B. Wu, Q. Wu, Z. Qiu, Z. Zhao and B. Z. Tang, Highly efficient circularly polarized near-infrared phosphorescence in both solution and aggregate, *Nat. Photonics*, 2024, **18**, 1276-1284.
9. L. Yao, G. Niu, J. Li, L. Gao, X. Luo, B. Xia, Y. Liu, P. Du, D. Li, C. Chen, Y. Zheng, Z. Xiao and J. Tang, Circularly Polarized Luminescence from Chiral Tetranuclear Copper(I) Iodide Clusters, *J. Phys. Chem. Lett*, 2020, **11**, 1255-1260.
